# Jiawei Suanzaoren decoction for the treatment of perimenopausal insomnia: clinical observation and experimental study

**DOI:** 10.3389/fphar.2024.1495957

**Published:** 2025-01-29

**Authors:** Li-Qiong Chen, Yong-Can Zhou, Fang Ning, Ming-Jin Zhu

**Affiliations:** ^1^ Department of Psychiatry, Tongde Hospital of Zhejiang Province, Hangzhou, China; ^2^ Department of Rehabilitation Medicine, Tongde Hospital of Zhejiang Province, Hangzhou, China

**Keywords:** Jiawei Suanzaoren decoction, perimenopausal insomnia, clinical observation, network pharmacology, experimental study

## Abstract

**Background:**

Traditional Chinese medicine has a good clinical therapeutic effect on perimenopausal insomnia, Jiawei Suanzaoren Tang (JW-SZRT) has a significant therapeutic effect on people with yin deficiency and fire excess type insomnia.

**Method:**

Firstly, 80 cases of perimenopausal yin deficiency and fire excess type insomnia were included in the study. The treatment group was treated with Jiawei Suanzaoren decoction, while the control group was treated with lorazepam for 4 weeks. The study observed and compared the Pittsburgh Sleep Quality Index (PSQI), Kupperman score, Polysomnography (PSG) and sex hormone levels of two groups before and after treatment. Secondly, we also conducted network pharmacology analysis and mechanism research. We first searched public databases for the targets of the compound known tobe associated with JW-SZRT, as well as those predicted to be targets of insomnia, and then used software Cytoscape 3.7.2, GO and KEGG to identify enriched gene pathways and networks. Networks and pathways that overlapped between Insomnia-associated proteins and JW-SZRT target proteins were then used to predict candidate protein targets of JW-SZRT in insomnia. Finally, 40 rats were selected in animal experimentation and 30 perimenopausal insomnia rat models were created through ovariectomy and a modified multi-platform water environment 72-hour sleep deprivation method. The model group received normal saline, the western medicine group received lorazepam suspension, and the Chinese medicine group received Jiawei Suanzaoren decoction. The sham surgery group was given routine feeding. The sleep condition, the levels of blood hormone levels and the expression levels of 5-Hydroxytryptamine (5-HT) and Gamma-Aminobutyric Acid receptor A (GABAA) receptor in the hypothalamus were compared between groups.

**Results:**

In clinical observation, the sleep condition and the changes of hormone levels between the treatment group and the control group were statistically significant (*P* < 0.05). The results of network pharmacology indicate that in the main signaling pathways for treating insomnia, it is speculated that Jiawei Suanzaoren Tang can regulate the signaling pathway of neuroactive ligand receptor interactions by inhibiting the activation of related proteins, thereby improving sleep. Animal experiments have also shown that the JW-SZRT can significantly improve the sleep conditions of insomnia rats during perimenopause, and the expression levels of 5-HT and GABAA receptors in the hypothalamus had a significant difference between groups in animal experiments (*P* < 0.05).

**Conclusion:**

Jiawei Suanzaoren Decoction can improve the perimenopausal insomnia. The mechanism behind this improvement may be related to the regulation of Estradiol (E2), Follicle-Stimulating Hormone (FSH), and luteinizing hormone (LH) levels, as well as the mRNA expression levels of 5-HT_1a_, 5-HT_2a_, GABAAR_α1_, and GABAAR_γ2_ receptors in the hypothalamus.

## 1 Introduction

The hypothalamus plays a role in regulating various physiological activities of the body, such as sleep and emotional regulation, through the endocrine system and autonomic nervous system (Sakurai, 2012). Hypothalamic neurons have estrogen receptors, which allow estrogen to counter-regulate them (Sá et al., 2015). This counter-regulatory effect can inhibit the secretion of gonadotropins in the body. After a woman enters perimenopause, the secretion of estrogen decreases, which weakens its counter-regulatory effect and reduces its inhibitory effect. This leads to an excess of gonadotropin produced by the pituitary gland, disrupting the automatic regulatory mechanism of the Hypothalamic pituitary ovarian (HPO) axis and upsetting the original balance between endocrine system (ES) and autonomic nervous system (ANS). During perimenopause, the body may experience a range of uncomfortable symptoms, including hot flashes, night sweats, sleep disturbances, and increased susceptibility to stress and anxiety. These symptoms are collectively known as perimenopausal syndrome.

A significant amount of estrogen receptors (ERα and ERβ) are present in the central nervous system. Estrogen exerts a significant role on the brain’s neurotransmitter systems, particularly the serotonin (5-HT) system, through estrogen receptors (ERα and ERβ).When estrogen levels decline, there is a decreasing trend in 5-HT_1a_ receptor levels, while estrogen negatively regulates the 5-HT_2a_ receptor through ERβ (Chhibber et al., 2017). When the expression level of the 5-HT_1a_ receptor gene increases in the brain, and the expression level of the 5-HT_2a_ receptor gene decreases, the 5-HT content increases. According to electroencephalogram analysis, an increase in the expression level of the 5-HT_1a_ receptor gene prolongs the time to fall asleep in REM sleep and shortens its duration (Monti and Jantos, 2003). An increase in the expression level of the 5-HT_2a_ receptor gene prolongs the duration of slow wave sleep and does not disturb the time and duration of rapid eye movement sleep. This leads to an increase in sleep depth and an improvement in sleep to a certain extent. Estrogen can bind to the G protein-coupled ER (GPR30) on the cell membrane, causing a non-genomic effect. GPR30 has a sedative effect to a certain extent, increases the expression level of GABAA receptor mRNA, promotes the conduction of the amino acid neurotransmitter GABA, and exerts an inhibitory effect. This improves the body’s state of excitement caused by anxiety (Tian et al., 2013). GABAA receptors, specifically the GABAAR_α1_ and GABAAR_γ2_ types, are commonly used indicators in insomnia research. When GABA, an amino acid neurotransmitter, binds to the R on the cell membrane, it opens the CI^−^ entry and exit channels, allowing a large amount of CI^−^ to enter the cell from outside. This strengthens the hyperpolarization state of the cell membrane potential, which is positive outside and negative inside, making it less likely for an occurrence to happen. Depolarization inhibits the generation of action potentials (Dellal et al., 2012). Therefore, GABA limits excitatory conduction and promotes sleep.

Suanzaoren Decoction is the preferred prescription for treating insomnia in traditional Chinese medicine. This topic combines “Suanzaoren Decoction” and “Zhizichi Decoction” to form the Jiawei Suanzaoren Decoction, which has an outstanding curative effect. Suanzaoren Decoction not only has sedative and hypnotic properties but can also regulate emotions. It is also effective in treating anxiety and convulsions. Its mechanism may be related to neurotransmitters and cytokines. Studies have shown that Suanzaoren Decoction’s regulatory effect on sleep is closely related to the amino acid neurotransmitter GABA, the monoamine neurotransmitter 5-HT, and their receptors. The water extract of Suanzaoren can act on the central nervous system through 5-HT1A, 5-HT2, GABA receptors (Liao J. F. et al., 1995). Suanzaoren Tang can treat insomnia by changing GABA content and regulating the expression of GABA receptors (Pei-Lu et al., 2007). Zhizichi Decoction is mainly used to treat insomnia caused by deficiency and restlessness. It has a calming effect on the mind and can enhance the sedative, hypnotic, and mood-regulating effects of Suanzaoren Decoction.

The aim of this study is to investigate the clinical effectiveness of Jiawei Suanzaoren Decoction in treating perimenopausal insomnia caused by yin-deficiency and fire-excess syndrome. This will be achieved by monitoring changes in sleep status and sex hormone levels. The perimenopausal insomnia model was created by bilateral ovarian removal and the water platform method. Sleep status can be evaluated by monitoring Sleep Onset Latency (SOL), Total Sleep Time (TST), and sleep onset rate. Changes in the content of three sex hormones can be detected using ELISA, while related genes in the hypothalamus of rats can be detected using Real Time-PCR. The expression of 5-HT and GABA receptor mRNA provides a foundation for in-depth research on the mechanism of Jiawei Suanzaoren decoction in treating perimenopausal insomnia.

## 2 Materials and methods

### 2.1 Patients

**FIGURE 1:**
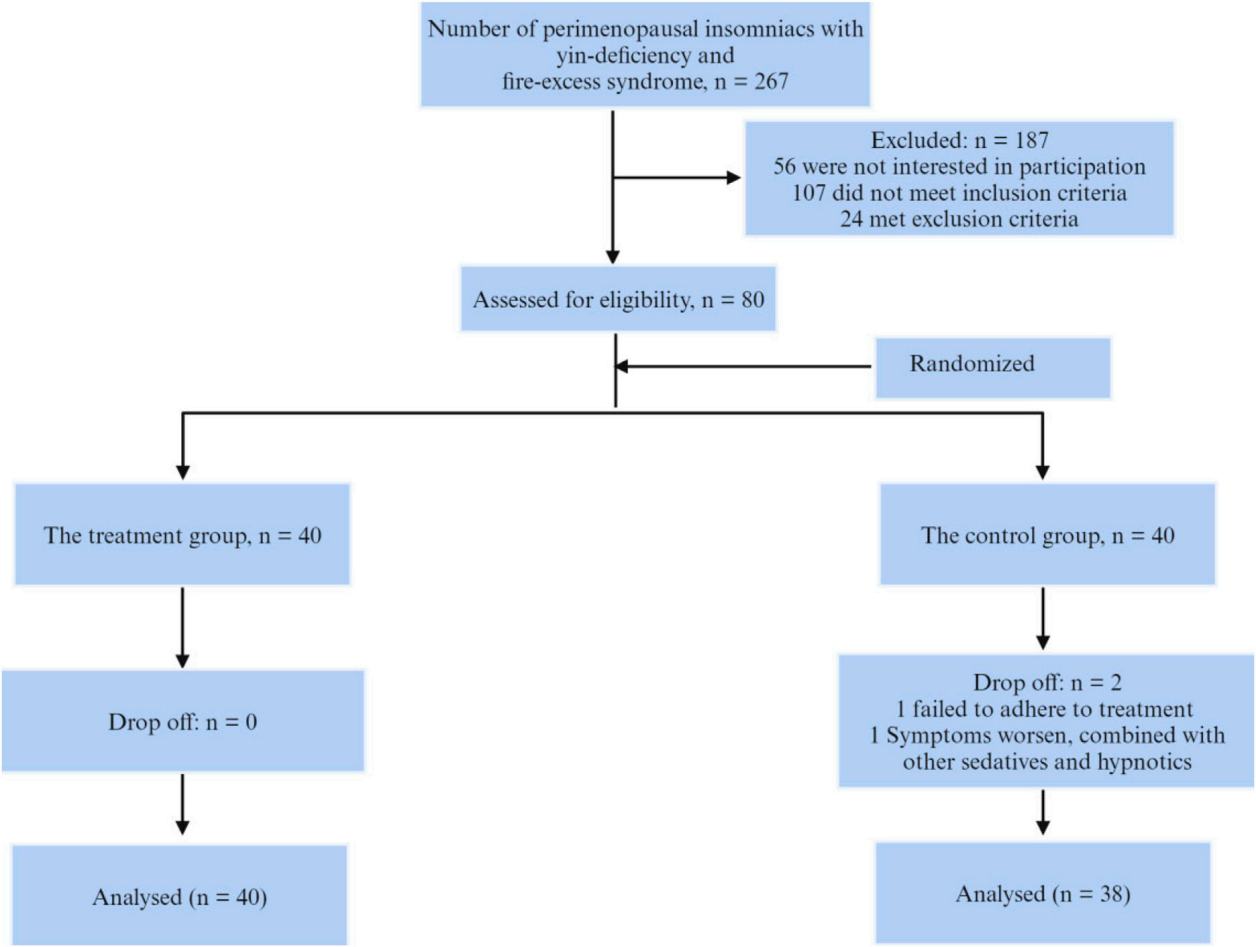
Flowchart of subject enrollment.

The study included 80 patients aged 40–60 years who were treated at Hangzhou Traditional Chinese Medicine Hospital from May 2019 to December 2019, and who met the diagnostic criteria for perimenopausal insomnia of yin deficiency and fire excess type. Inclusion criteria were as follows: (a) fulfilling the diagnostic criteria for perimenopausal syndrome according to “Obstetrics and Gynaecology” (2000), “Chinese Obstetrics and Gynaecology” (1999) and “Guiding Principles for Clinical Research of New Traditional Chinese Medicines” (2002). (b) fulfilling the diagnosis of chronic insomnia according to the DSM-5; (c) yin deficiency and fire excess syndrome, referring to the classification criteria for “insomnia” with yin deficiency and fire excess syndrome in the “Diagnostic and Therapeutic Criteria for Traditional Chinese Medicine Diseases” (1994); (d) having an educational level of junior high school or above. Exclusion criteria included: (a) comorbidity with other mental illnesses; (b) use of drugs that affect the concentration of sex hormones in the body in the past month; (c) history of use of other sedative-hypnotic and other psychiatric drugs in the past month; (d) other organic diseases that affect diagnosis. For example, endocrine disorders (hyperthyroidism or hypothyroidism, *etc.*), neurological disorders (stroke, Alzheimer’s disease, Parkinson’s disease, *etc.*), insomnia caused by drugs or substances (caffeine, nicotine, antidepressants, antihypertensive drugs, *etc.*); (e) those who refuse to cooperate and it is difficult to determine the therapeutic effect. This research protocol was reviewed and approved by the Medical Ethics Committee of Hangzhou Hospital of Traditional Chinese Medicine (approval number: 2019KY024). All patients signed a written informed consent and were willing to undergo follow-up. The subject enrolment process is shown in Figure 1.

### 2.2 Treatment

80 patients who meet the inclusion criteria will be randomly divided into the treatment group (TG; n = 40) and the control group (CG; n = 40) in a 1:1 ratio using computer-generated random numbers. The control group took oral lorazepam tablets (Lola, 0.5 mg/tablet, registration number: H20130909, manufactured by Hainan Atlantic Pharmaceutical Factory Co., Ltd.), one tablet each 30 min after breakfast and 30 min before bedtime, for 1 month. The treatment group was treated with Jiawei Suanzaoren decoction (Suanzaoren, Fuling, Chuanxiong, Zhimu, Zhizi, Dandouchi and Gancao, ratio 1.5:1.5:1:1.2:1:1:1:0.5). In Table 1. Purchased from the Traditional Chinese Medicine Pharmacy of the Hangzhou Traditional Chinese Medicine Hospital, each dose is decocted into 400 mL and divided into 2 packets (taken at 2 pm and 1 h before bedtime) for 4 weeks. All subjects were forbidden to drink tea, coffee, alcohol and cigarettes throughout the study period to avoid their potential effect on treatment efficacy.

**TABLE 1:** The ingredients of the Jiawei Suanzaoren decoctioncan.

Pharmaceutical name	Chinese name	Latin botanical name	Proportion (%)
Semen ZizyphiSpinosae	Suanzaoren	Ziziphus jujuba Mill. var. Spinosa	19.48
Sclerotium Poriae Cocos	Fuling	Poria cocos (Schw.) Wolf	19.48
Radix LigusticiChuanxiong	Chuangxiong	Ligusticum chuanxiong Hort	12.99
RhizomaAnemarrhena	Zhimu	AnemarrhenaasphodeloidesBge	15.58
Gardenia Jasminoides fruit	Zhizi	Gardenia jasminoides Ellis	12.99
Fermented Soybean	Dandouchi	Semen SojaePraepatum	12.99
Radix Glycyrrhizae	Gancao	Glycyrrhiza uralensis Fisch	6.49

Patients receiving the intervention and investigators are not affected by the treatment allocation. However, outcome assessors and statisticians are blind to the group allocation.

During the entire study period, 2 cases were excluded from the control group, 1 case was treated with traditional Chinese medicine due to the patient’s refusal to take medication, and 1 case was treated with a combination of other sedatives and hypnotics due to worsening symptoms; the treatment group completed the entire study.

### 2.3 Efficacy evaluation

The PSQI scale consists of two parts, one part (19 items) is self-rated and the other part (5 items) is scored with the help of others. Among these, the scores of the 19th self-assessment item and 5 other scored items are not included in the total score, and the remaining 18 items are divided into 7 parts. The scores are based on specific conditions and are assessed once before and once after treatment. The higher the score, the more attention the sleep problem deserves. The Kupperman Score is commonly used clinically to assess a patient’s perimenopausal symptoms and severity, the higher the score, the more severe the condition. Sleep stage scores (30 s per stage) were performed on all subjects’ sleep recordings at baseline and week 4 of treatment by a registered PSG technology expert. PSG sleep data includes total sleep time (TST), latency to onset of persistent sleep (LPS), wake after sleep onset (WASO), slow wave sleep (SWS), and rapid eye movement (REM) sleep.

### 2.4 Blood E_2_, FSH and LH levels are determined by ELISA

To compare the changes in blood hormone levels before and after treatment between the treatment group and the control group.

### 2.5 Clinical efficacy

The clinical effectiveness of Western medicine is assessed using the PSQI score reduction rate (the percentage difference between the total score before and after treatment and the total score before treatment). The details are as follows: (a) a score reduction rate greater than 75% indicates clinical recovery; (b) a score reduction rate between 50% and 74% indicates significant clinical efficacy; (c) a score reduction rate between 25% and 49% indicates clinical effectiveness; (d) a score reduction rate less than 25% indicates clinical ineffectiveness.

### 2.6 Adverse reactions

Use the Treatment Emergent Symptom Scale (TESS) to count abnormalities experienced by patients during the trial: excessive sleep, abnormal gastrointestinal function, liver damage, headache, etc. and record them at any time.

### 2.7 Network pharmacology

Collect all chemical components of seven medicinal herbs, namely, “Suanzaoren”, “Zhimu”, “Chuanxiong”, “Fuling”, “Gancao”, “Dandouchi”, and “Zhizi”, from the Traditional Chinese Medicine Systems Pharmacology Database and Analysis Platform (TCMSP) of Jiawei Suanzaoren Tang. Set the conditions of “OB ≥ 30%” and “DL ≥ 0.18” simultaneously to screen the chemical components of Jiawei Suanzaoren Tang for activity, obtain the relevant targets corresponding to the active ingredients of Jiawei Suanzaoren Tang, use Perl software scripts to filter the active ingredients in batches, screen out the potential target information corresponding to each active ingredient, and obtain the full name of the target gene. With the help of the Uniprot database, the species was set as *Homo sapiens*, and all the targets of the active ingredients in Jiawei Suanzaoren Tang were corrected to gene names (Symbols) to solve the problem of non-standard drug target names. Construct a protein protein interaction network (PPI network) using Cytoscape software, and perform network topology parameter analysis and calculation by Network Analyzer Plugin to obtain network information such as Degree, BC, and CC. Further set the node size and color reflects the degree value size, and the edge thickness reflects the combined score size, ultimately obtain the protein-protein interaction (PPI) network. Search for keywords such as “Insomnia”, “Sleep Disorder”, “Sleep Disorders”, “Sleeplessness”, “Agrypnia” in GeneCards database, OMIM database, mala database, and CTD database to find relevant genes, remove duplicate and false positive genes, and obtain a dataset of predicted target information for insomnia. Next, use the Venny online tool to draw a Venny diagram of the active ingredient targets and insomnia targets of Jiawei Suanzaoren Tang, and obtain the intersection targets. Import the intersection target dataset of Jiawei Suanzaoren Tang and insomnia disease into the String database, limiting the research species to *Homo sapiens*. In the String database, the default minimum required interaction score is 0.400, and the PPI network is obtained. Analyze the core genes of the PPI network using R script. Perform GO and KEGG enrichment analysis in DAVID.

### 2.8 Experimental animal

Healthy female SPF grade SD rats, weighing approximately (200 ± 20) g. Purchased from the Animal Centre of Zhejiang University of Traditional Chinese Medicine, with animal certificate number: SYXK (Zhejiang) 2018-0012. The experimental rats were kept in a temperature of 23°C ± 2°C and humidity of 55% ± 15%, with 12 h of light per day to ensure food and water supply. The feeding environment was cleaned in a timely manner and regularly disinfected and sterilised. Forty experimental rats were sequentially measured for body weight, tagged with ear tags and randomly divided into sham surgery group, model group, traditional Chinese medicine group and western medicine group, with 10 rats in each group, and acclimated for 7 days.

### 2.9 Moulding

Modelling methods for perimenopausal models include: castration surgery, natural ageing (Hu et al., 2015), stress, radiation, drugs and metabolites of 4-vinylcyclohexene can damage the ovaries and cause ovarian failure. Total castration is simple and widely used (Shi et al., 2015). Establishment of an animal model of perimenopausal insomnia caused by ovariectomy and improved small platform water environment sleep deprivation method (Zhang et al., 2023; Dai et al., 2021). Female rats were weighed, routinely anaesthetised and prepared for pelt preparation. The skin and muscles were incised 3.5 cm from the vaginal opening in the lower mid-abdomen to locate the oviducts and ovaries. The fallopian tubes were routinely ligated and the fatty area at the end of the tubes, the ovaries, was removed. The wounds were sutured in layers, disinfected with iodine and anti-inflammatory penicillin to establish a perimenopausal model. After 10 days, an animal insomnia model was established using a modified horizontal table method. The rat is placed in an aquarium containing 4 columns (platform diameter: 7 cm, 2 cm above the water level). Food and water are provided *ad libitum*. As soon as the rats fall asleep, they suffer from insomnia due to muscle tension.

Beginning 5 days after surgery, take vaginal secretion smears from perimenopausal rats. Hold the rat in a supine position with your left hand, hold a cotton swab with your right hand, moisten it with a little physiological saline (NS) and rotate it twice from the vaginal opening into the vagina to remove it. Roll gently from left to right on the surface of the slide. Allow to stand for 5 min, stain with haematoxylin and eosin and air dry naturally. If the oestrus cycle is not observed, this indicates that the perimenopausal model is well made. Whether the insomnia model is successfully modelled can be assessed by sleep experiments. Rats were intraperitoneally injected (i.p.) with pentobarbital sodium at a dose of 30 mg/kg and specific parameters such as sleep onset time (sleep: rest, no reset reflex, lasting more than 1 min), sleep duration (time from entry into sleep to awakening, with 3 turns within 30 s) and sleep onset rate (number of sleeps within 10 min) were monitored. After modelling, the rats showed prolonged sleep duration, shortened sleep duration and daytime arousal, indicating successful establishment of the model.

### 2.10 Gavage

The model group received 20 mL/kg/d of normal saline, the Western medicine group received 0.2 mg/kg/d of lorazepam suspension (concentration 0.01 mg/mL), and the Chinese medicine group received 10 g/kg/d of modified sour jujube seed soup (concentration 0.5 g/mL). The Chinese medicine group received routine feeding, with 2 mL per dose administered by gavage in the morning and evening (8:00 a.m., 4:00 p.m.) for 14 consecutive days.

### 2.11 Sampling and testing

Inject 7% chloral hydrate (350 mg/kg, i.p.) into the abdominal cavity. After the anaesthetic has taken effect, cervical dislocation occurs immediately and the patient is euthanised with minimal pain; rapidly puncture the femoral artery to collect blood, remove brain tissue and prepare a sample. Blood samples: Separate blood, preserve plasma and serum samples. Brain tissue samples: Quickly dissect and classify brain tissue (such as thalamus), freeze on dry ice and store in a −80°C freezer for testing. Use ELISA to detect levels of E_2_, FSH and LH in the serum of perimenopausal insomnia rats and Real Time PCR to detect mRNA levels of 5-HT_1a_ receptor, 5-HT_2a_ receptor, GABAAR_α1_ and GABAAR_γ2_ in the hypothalamus. The details are as follows: (a) Using β-actin as an internal reference, the literature was searched to determine the cDNA sequences of 5-HT_1a_ receptor, 5-HT_2a_ receptor, GABAAR_α1_ and GABAAR_γ2_. Primer sequences are listed in Table 2. (b) Extract total RNA according to the instructions of the miRNeasy Mini Kit and measure RNA concentration and purity using a nucleic acid protein analyser; (c) Place the extracted RNA, cDNA reverse transcription reagents and enzyme-free sterile hydrolysate on ice. Configure the required reverse transcription reaction system according to Table 3, with a total volume of 20 μL, −20°C low temperature storage for backup. (d) Configure the real-time PCR reaction system according to Table 4, with a total volume of 10 μL. Each group was subjected to 3 replicate wells of real-time PCR amplification and the relevant parameters are shown in Table 5.

**TABLE 2:** Primer sequence.

Receptor subtypes	Primer sequence	Length
5-HT_1a_	5′- TCA​CCT​GCG​ACC​TGT​TTA​TC-3′	394 bp
	5′- GCT​CCC​TTC​TTT​TCC​ACC​TT-3′	
5-HT_2a_	5′- CAT​GCC​TCT​CCA​TTC​TTC​ATC​TCC​AGG​AA-3′	611 bp
	5′-CAA​GGT​GGC​TTC​TTT​CTG​AAG​TGA​CTT​GA-3′	
GABA_A_R_α1_	5′-AGT​GTG​CTA​TGC​CTT​CGT​GTT​C-3′	246 bp
	5′-ACT​TCT​TTC​GGT​TCT​ATG​GTC​G-3′	
GABA_A_R_γ2_	5′-ACG​ACA​CCA​CCA​CCG​ACA​AC-3′	247 bp
	5′-TGG​ATC​AGA​AAC​TGG​GAC​AAG​G-3′	
β-actin	5′- TGG​TGG​GTA​TGG​GTC​AGA​AGG​ACT​C-3′	265 bp
	5′- CAT​GGC​TGG​GGT​GTT​GAA​GGT​CTC​A-3′	

**TABLE 3:** Reverse transcription steps.

Reagent	Amount of usage
Total RNA	500 ng
Primer	1 μL
RNase Free dH_2_O	Up to 12 μL
65°C, 5 min	
5*reaction buffer	4 μL
10 mM dNTP Mix	2 μL
RI	1 μL
RT	1 μL
42°C, 60 min	
75°C, 5 min	
4°C∞	

**TABLE 4:** Main reagents of the realtime PCR reaction system.

Reagent	10 μL reaction system
2*SYBR Green	5 μL
Primer mix	0.5 μL
cDNA	0.5 μL
RNase-free Water	Up to 10 μL

**TABLE 5:** Relevant parameters for realtime PCR amplification.

Step 1	50°C, 2 min
Step 2	95°C, 2 min
Step 3	95°C, 15 s
Step 4	60°C, 30 s
Step 5	72°C, 30 s Plate read
	Go to step 3, 40 cycles
Melt-curve analysis	65°C–99°C, 0.5°C/read

### 2.12 Statistical methods

All results were organised using SPSS 22. Age, PSQI score, Kupperman score, PSG monitoring results and hormone levels between two groups are quantitative data. Paired t-tests were used to compare age between the two groups. Paired t-tests are used to compare PSQI score, Kupperman score, Difference in PSG monitoring results and hormone levels between the two groups and before and after treatment; comparison of educational background and clinical effectiveness of Western medicine between the treatment group and the control group was done using Mann Whitney U test; comparison of adverse reactions between the treatment group and the control group was done using Chi-squared test. Weight, sleep time, hormone levels and gene expression levels of rats are expressed as mean plus minus standard deviation. Paired t-tests were used to compare weight before and after modelling, weight before and after administration, sleep time, sleep duration and hormone levels; to compare weight, sleep time, sleep duration, hormone levels and gene expression levels between multiple groups by analysis of variance; and to compare sleep rate by chi-squared test. Probability (P) values <0.05 were considered statistically significant.

## 3 Results

### 3.1 Demographic characteristics

There was no significant difference in baseline levels of age and educational attainment between the treatment and control groups (*P* > 0.05). In Table 6.

**TABLE 6:** Demographic characteristics of the participants.

Characteristics		TG	TG	t/Z	P
Age (years)		48.95 ± 2.45	47.82 ± 2.87	1.88	0.06
Education (cases)	Junior high school	5	8	−1.863	0.06
	High school	15	19		
	University	18	10		
	Graduate or above	2	1		

TG, the treatment group; CG, the control group.

### 3.2 Comparison of PSQI, Kuperman score, and PSG monitoring results

Before treatment, there was no statistical difference in sleep scores between the treatment group and the control group (*P* > 0.05). After treatment, compared to before treatment in this group, the sleep indicators of the treatment group and the control group decreased significantly (*P* < 0.05). Compared with the control group, the total score, sleep quality, sleep duration and sleep efficiency scores of the treatment group decreased significantly after treatment, and the differences were statistically significant (*P* < 0.05). In Table 7.

**TABLE 7:** Comparison of PSQI scores before and after treatment between two groups.

Factors	TG (n = 40)		CG (n = 38)	
	Before treatment	After treatment	Before treatment	After treatment
Total score	14.91 ± 1.35	8.19 ± 1.59^*#^	14.56 ± 1.14	9.54 ± 1.67^*^
Sleep quality	2.47 ± 0.58	1.24 ± 0.58^*#^	2.48 ± 0.47	1.52 ± 0.63^*^
Sleep latency	2.64 ± 0.55	1.89 ± 0.75^*#^	2.64 ± 0.44	2.29 ± 0.69^*^
Sleep time	2.37 ± 0.51	1.58 ± 0.62^*^	2.45 ± 0.53	1.46 ± 0.75^*^
Sleep efficiency	2.72 ± 0.43	1.17 ± 0.81^*#^	2.62 ± 0.45	1.72 ± 0.53^*^
Sleep disorders	2.52 ± 0.62	1.08 ± 0.49^*^	2.24 ± 0.74	1.37 ± 0.80^*^
Daytime function	2.19 ± 0.49	0.98 ± 0.50^*^	2.14 ± 0.55	1.19 ± 0.64^*^

Data were expressed as mean ± SD. TG, the treatment group, CG, the control group. *P < 0.05 when compared to pre-treatment in the same group. #*P* < 0.05 when compared with CG at the same observation point.

**FIGURE 2:**
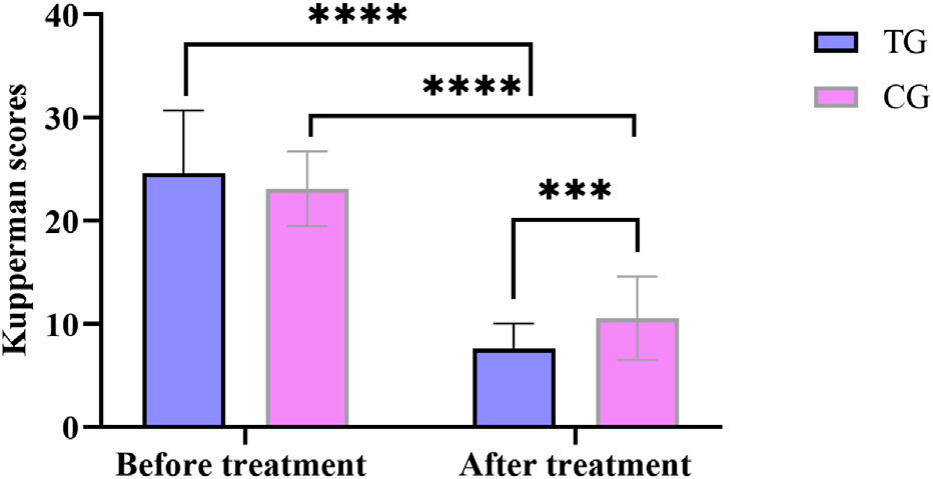
Comparison of Kupperman scores before and after treatment between the treatment group and the control group. All data was shown as mean ± SD.

Before treatment, there was no significant difference in total Kupperman scores between the treatment group and the control group (*P* > 0.05). After treatment, compared with the pre-treatment group, the total scores of the treatment and control groups decreased significantly (*P* < 0.05). Compared with the control group, the total score of the treatment group decreased significantly after treatment, and the difference was statistically significant (*P* < 0.05). In Figure 2.

**FIGURE 3:**
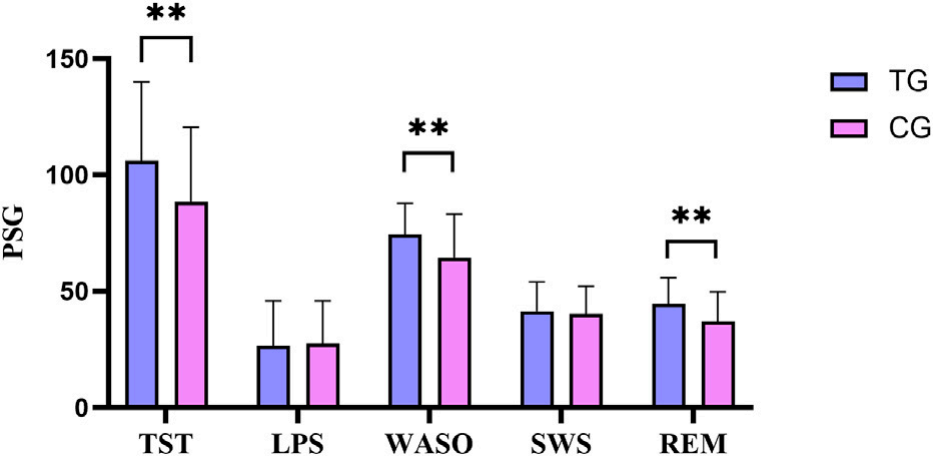
Comparison of PSG difference between the treatment group and the control group before and after treatment. All data was shown as mean ± SD.

Compared with the control group, the differences in TST, WASO, and REM before and after treatment were more significant in the treatment group, and the differences were statistically significant (*P* < 0.05). In Figure 3.

### 3.3 Serum indexes analysis

**FIGURE 4:**
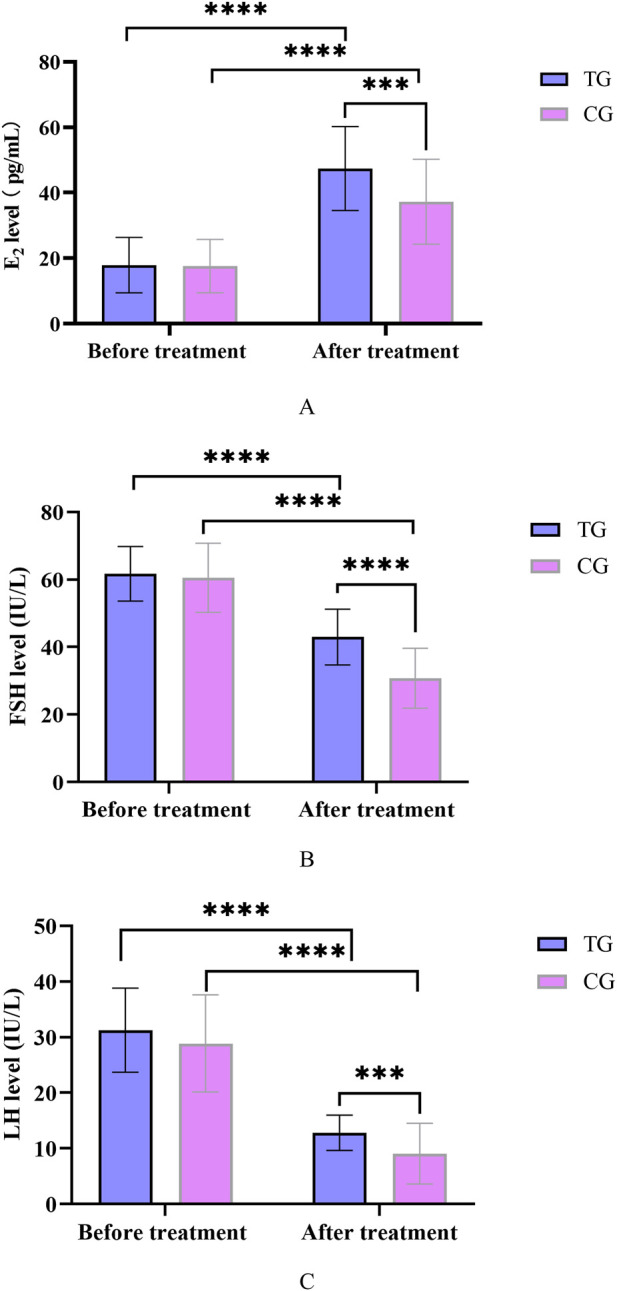
**(A)** Changes in E_2_ levels before and after treatment in TG and CG; **(B)** Changes in FSH levels before and after treatment in TG and CG; **(C)** Changes in LH levels before and after treatment in TG and CG. All data was shown as mean ± SD.

Before treatment, there was no significant difference in E_2_, FSH and LH levels between the treatment and control groups (*P* > 0.05). After treatment, compared with before treatment in this group, the E_2_ content of the treatment group and the control group was significantly increased (*P* < 0.05), and the FSH and LH levels were significantly decreased (*P* < 0.05). Compared with the control group, the E_2_ content of patients in the treatment group increased significantly, and the FSH and LH levels decreased significantly after treatment, and the difference was statistically significant (*P* < 0.05). In Figure 4.

### 3.4 Comparison of clinical efficacy

After treatment, the overall clinical efficacy of Western medicine was higher in the treatment group than in the control group, and there was a statistical difference (*P* < 0.05). In Table 8.

**TABLE 8:** Clinical efficacy of Western medicine (PSQI score reduction rate) (cases, %).

Group	n	Clinical recovery	Significant clinical efficacy	Clinical effectiveness	Clinical ineffectiveness	Total effective rate (%)	Z value	*P* value
TG	40	0	15	23	2	38 (95)		
CG	38	0	5	25	8	30 (78.95)	−2.913	0.004

### 3.5 Comparison of adverse reactions

There was no significant difference in abnormal reactions between the treatment group and the control group (*P* > 0.05). In Table 9.

**TABLE 9:** Adverse reactions (examples, %).

Group	n	Drowsiness	Diarrhea	Nausea and vomiting	Dizzy	Total (%)	x^2^ Value	P value
TG	40	2	1	1	1	4 (10)	2.627	0.105
CG	38	4	1	2	8	9 (23.68)		

### 3.6 Network pharmacology results

**FIGURE 5:**
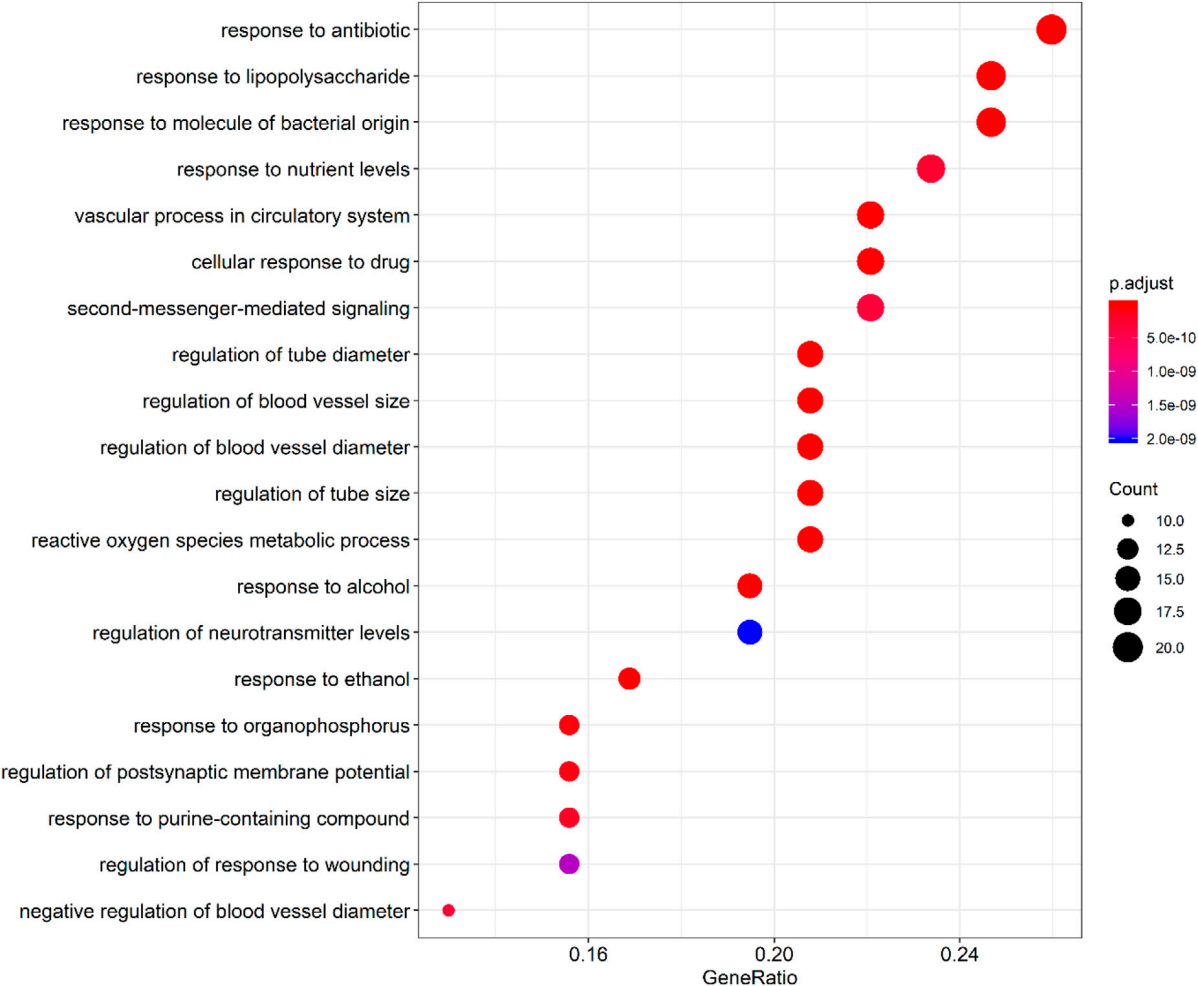
Bubble plot of GO-BP enrichment analysis.

**FIGURE 6:**
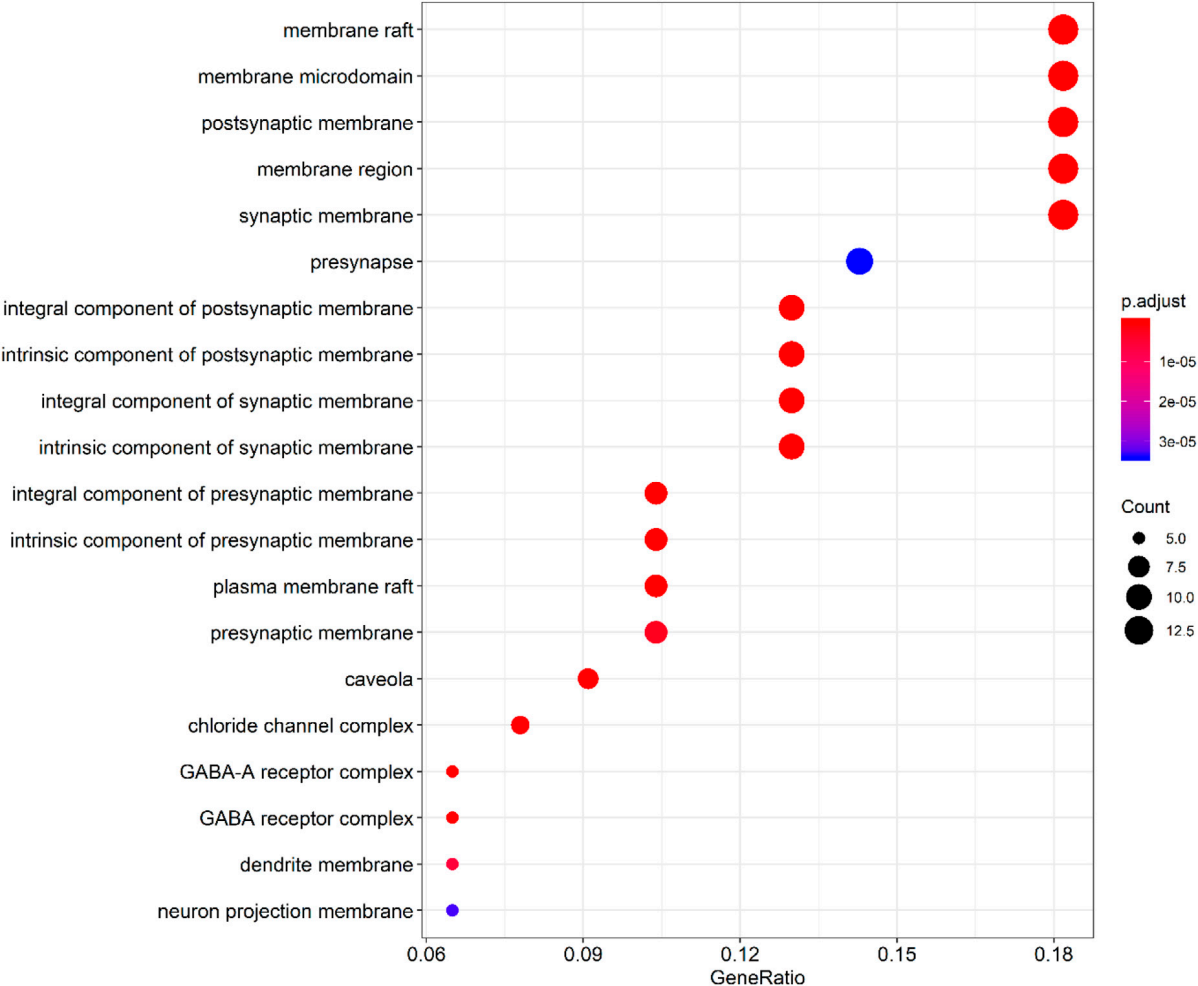
Bubble plot of GO-CC enrichment analysis.

**FIGURE 7:**
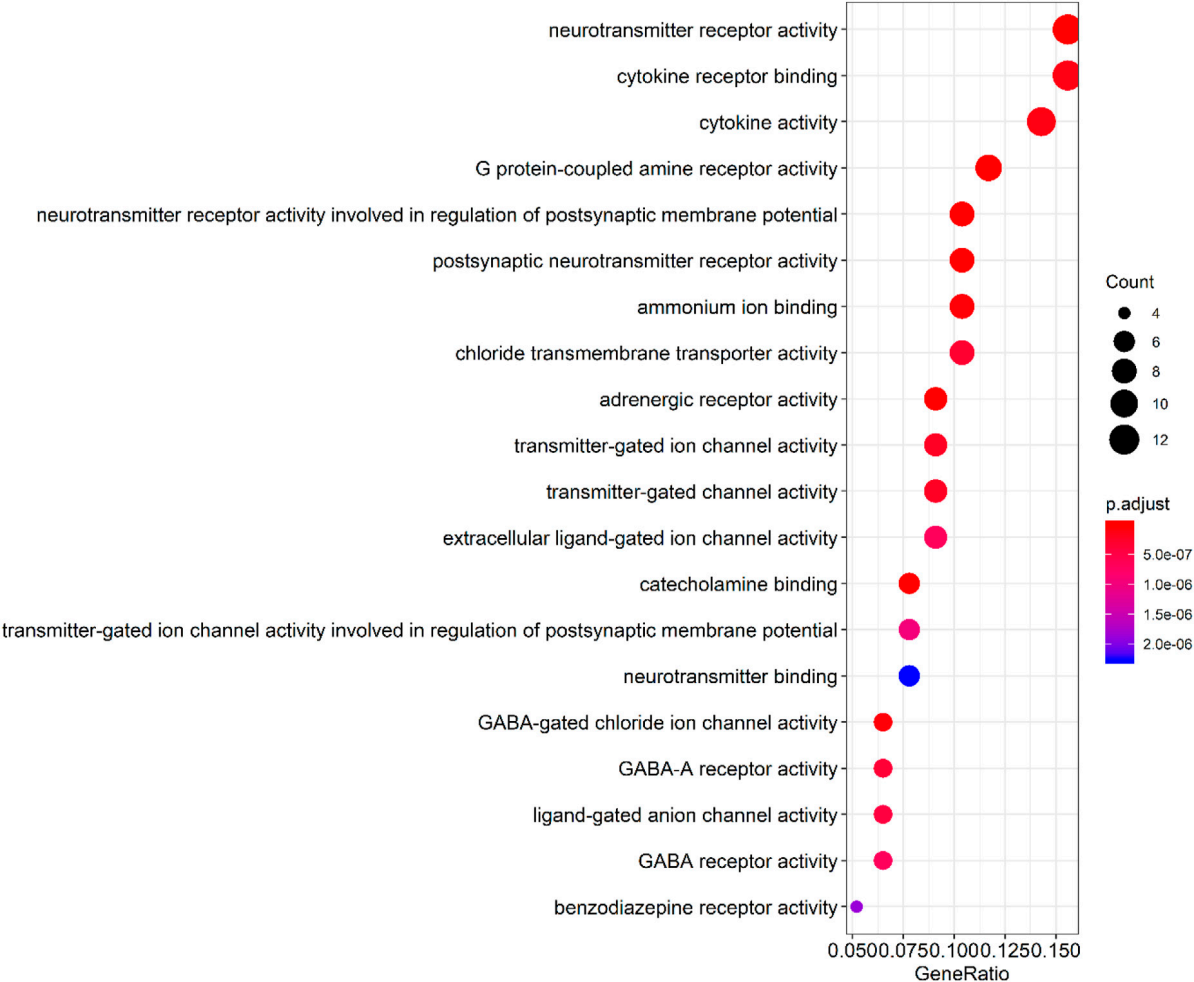
Bubble plot of GO-MF enrichment analysis.

**FIGURE 8:**
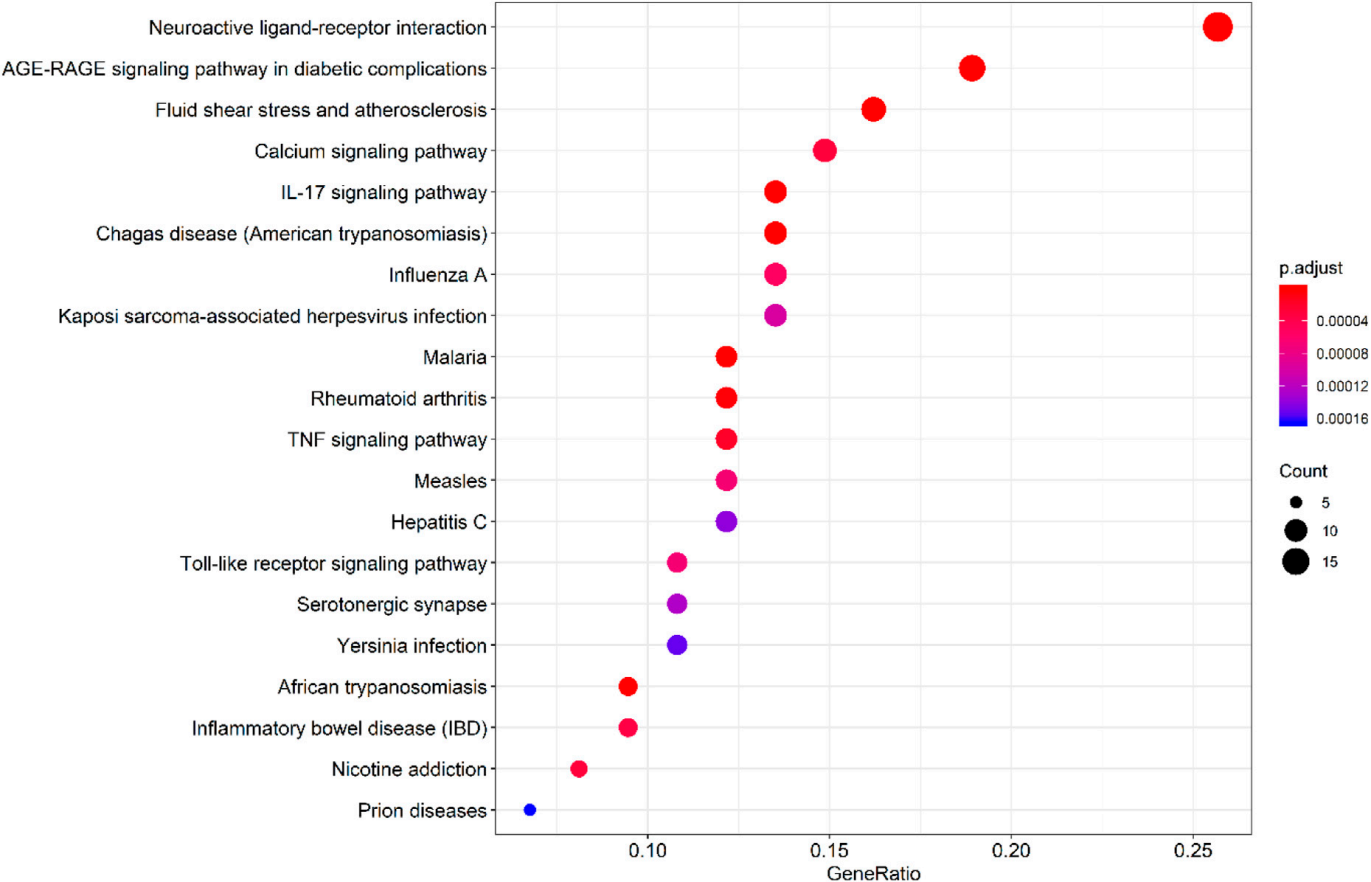
KEGG pathway enrichment analysis bubble plot of Jiawei Suanzaoren Decoction in treating insomnia targets.

**FIGURE 9:**
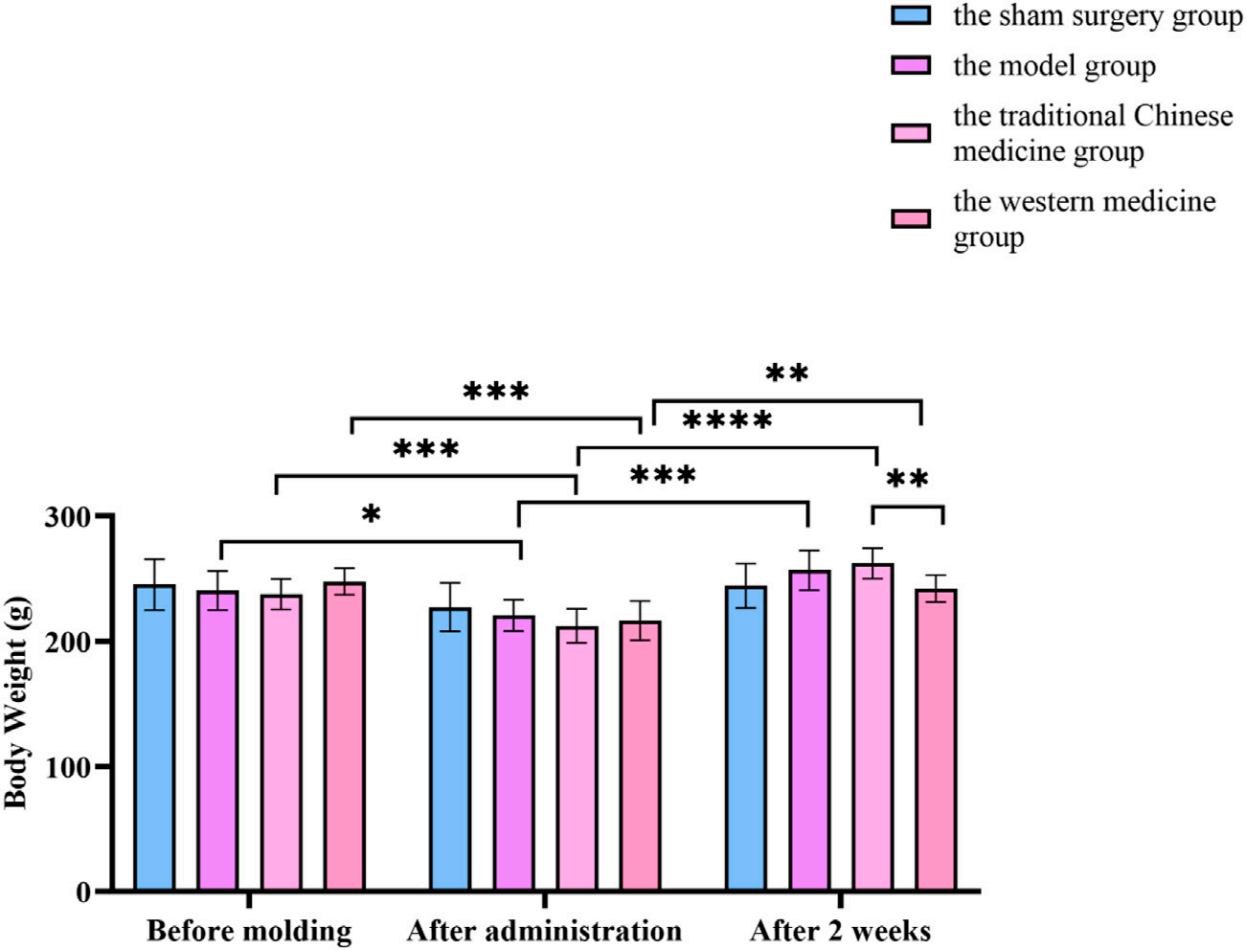
Changes in body weight before molding, after administration and after 2 weeks of treatment in the sham surgery group, the model group, the traditional Chinese medicine group and the western medicine group. All data was shown as mean ± SD.

**FIGURE 10:**
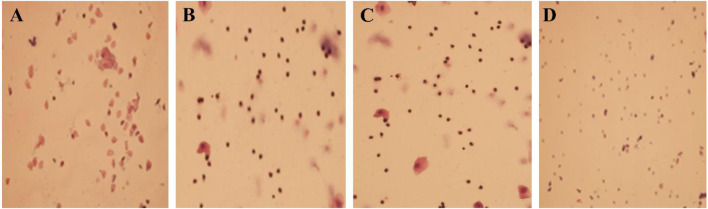
**(A)** The sham surgery group (×100); **(B)** the model group (×100); **(C)** the traditional Chinese medicine Group (×100); **(D)** the western medicine group (×100).

**FIGURE 11:**
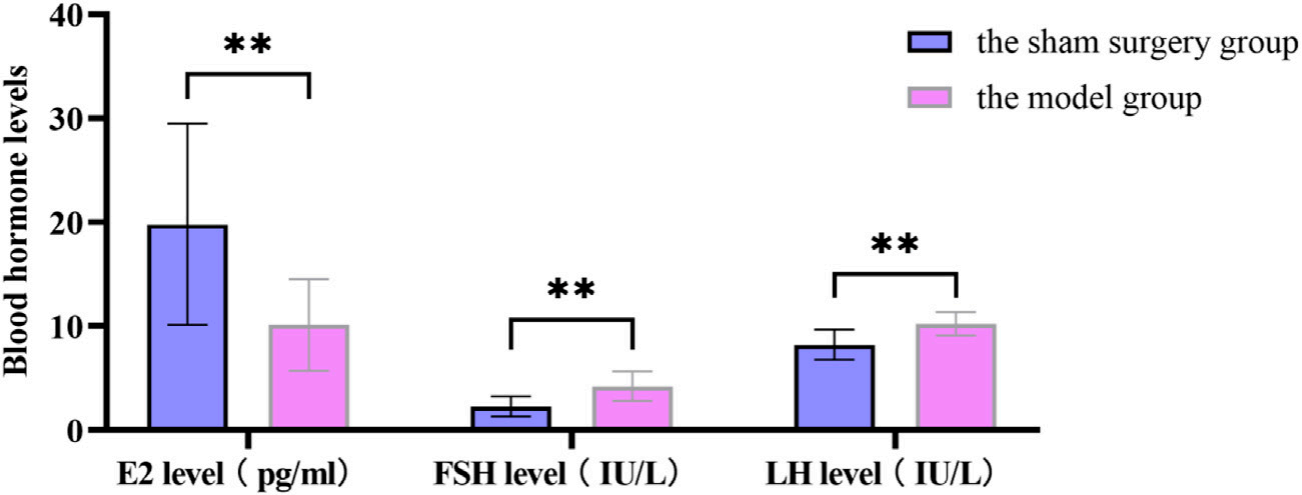
Changes in blood hormone levels between the sham surgery group and the model group rats. All data was shown as mean ± SD.

**FIGURE 12:**
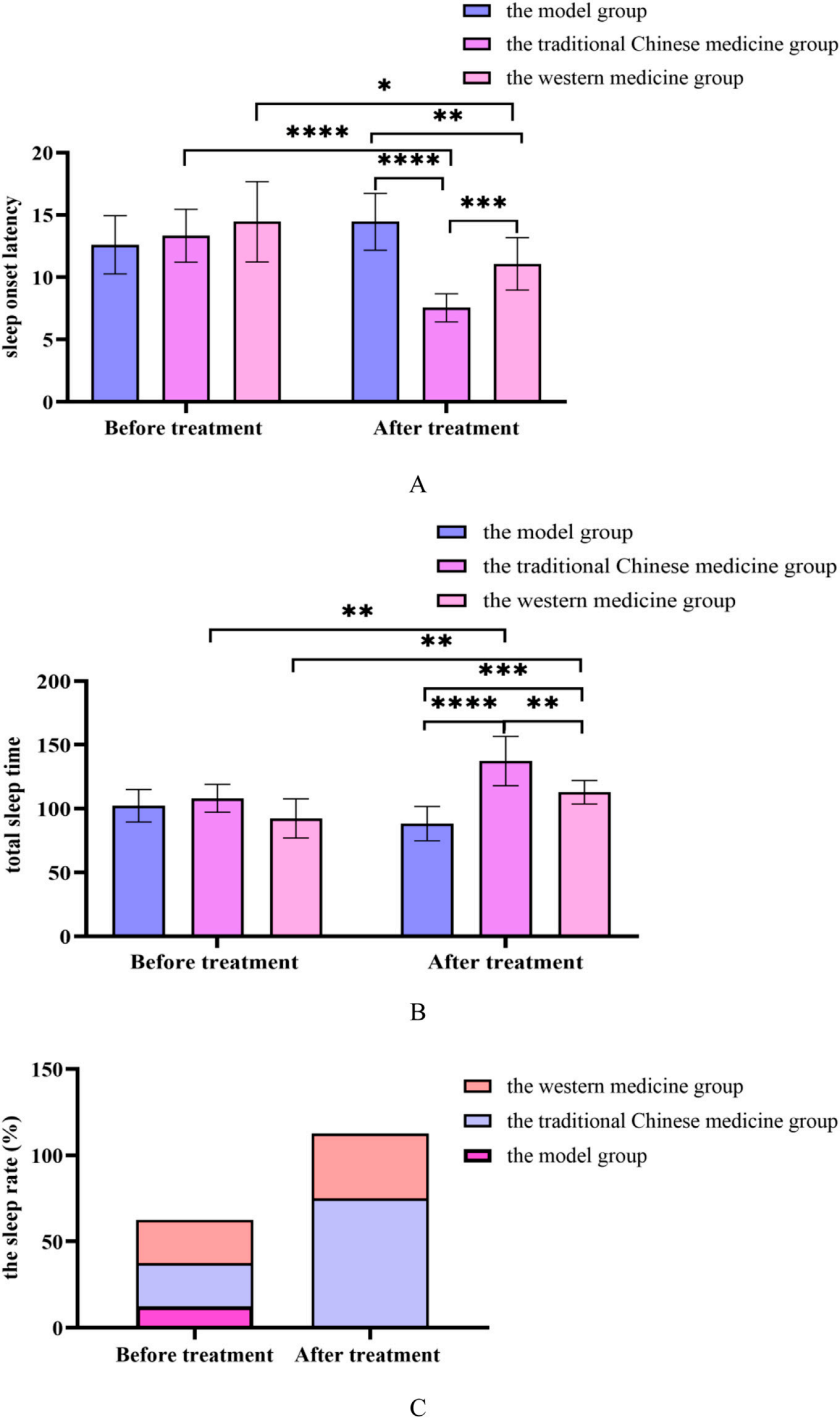
**(A)** Changes in the sleep onset latency before and after treatment in the model group, the traditional Chinese medicine group and the western medicine group; **(B)** Changes in the total sleep time before and after treatment in the model group, the traditional Chinese medicine groupand the western medicine group; **(C)** Changes in the sleep rate before and after treatment in the model group, the traditional Chinese medicine groupand the western medicine group. All data was shown as mean ± SD.

**FIGURE 13:**
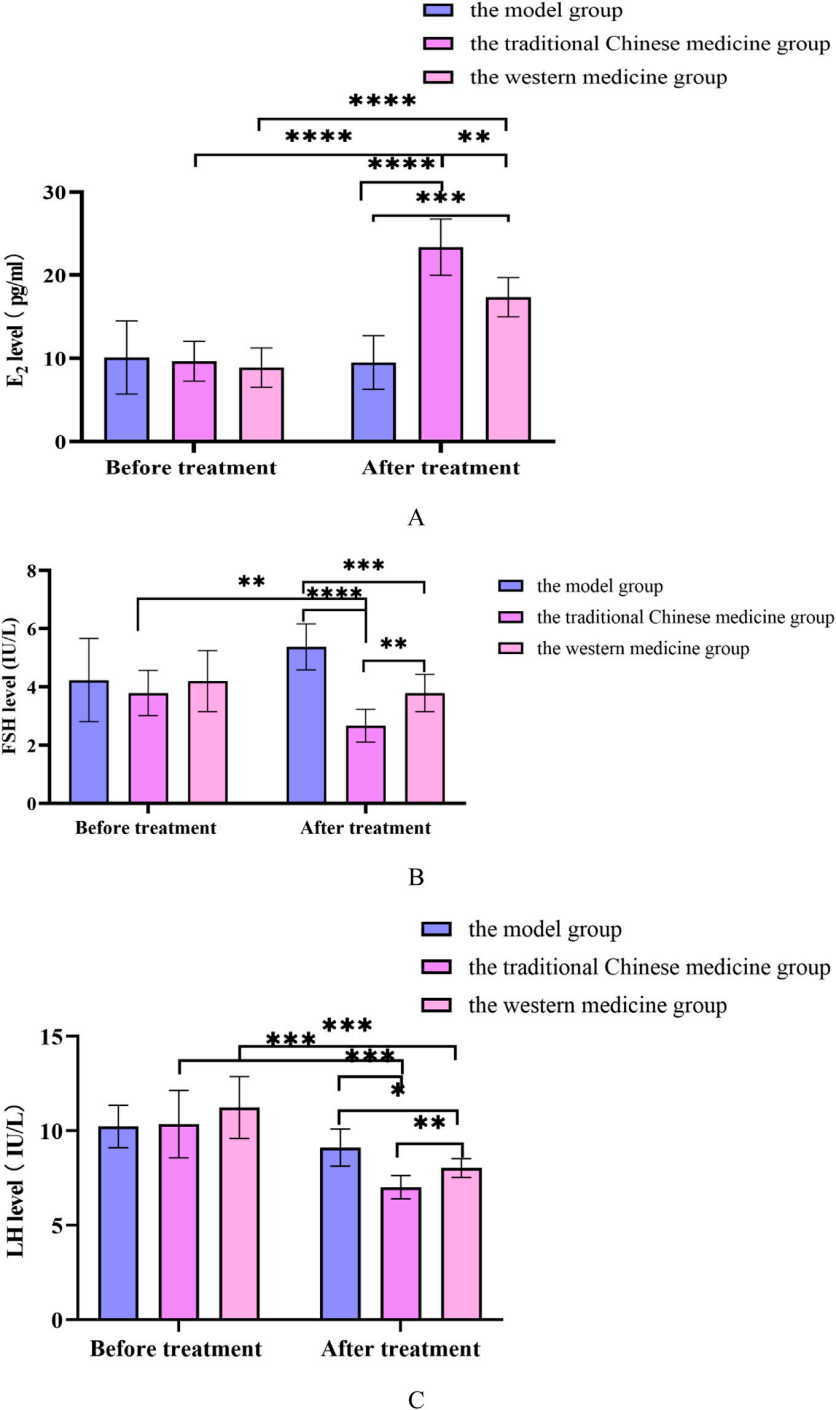
**(A)** Changes in E_2_ levels before and after treatment in the model group, the traditional Chinese medicine group and the western medicine group; **(B)** Changes in FSH levels before and after treatment in the model group, the traditional Chinese medicine group and the western medicine group; **(C)** Changes in LH levels before and after treatment in the model group, the traditional Chinese medicine group and the western medicine group. All data was shown as mean ± SD.

**FIGURE 14:**
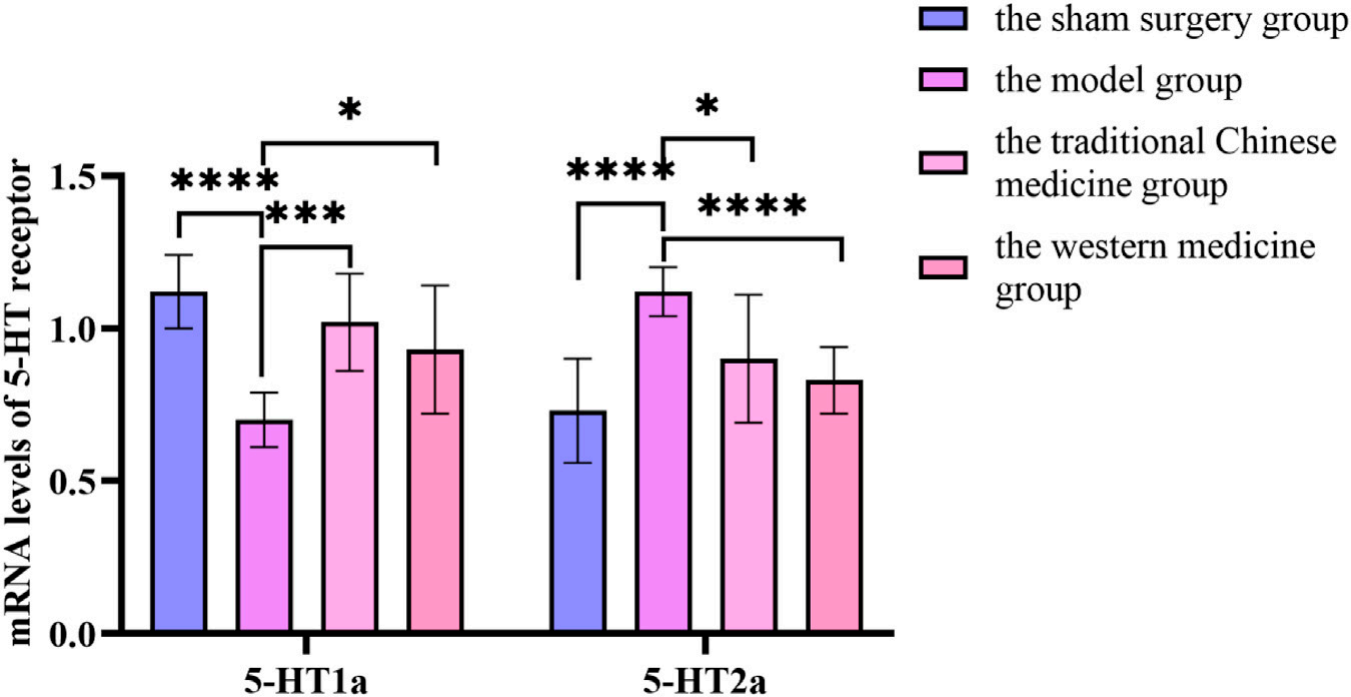
Changes in mRNA levels of 5-HT receptor before and after treatment in the sham surgery group, the model group, the traditional Chinese medicine group and the western medicine group.

By querying the TCMSP database for the chemical components of 7 traditional Chinese medicines contained in Jiawei Suanzaoren Tang, 155 potential active ingredients were screened based on the conditions of “OB ≥ 30%” and “DL ≥ 0.18”, and the potential target information corresponding to each active ingredient was obtained. After Perl program screening, a total of 2,430 effective ingredient targets were obtained, followed by 1832 symbols obtained through Uniprot. Using Cytoscape software to construct a relationship network between the active ingredients and targets of Jiawei Suanzaoren Tang. In Figure 5. 1931 insomnia related target information were obtained using public databases, and a network relationship diagram was constructed using Cytoscape software for the active ingredients target insomnia disease of Jiawei Suanzaoren Tang. In Figure 6. Using Venny 2.1, draw a Venny diagram of the active ingredients of Jiawei Suanzaoren Tang and the targets of insomnia, with a total of 77 intersecting targets. In Figure 7. Enter the String database to construct the interaction between the active ingredients and targets of Jiawei Suanzaoren Tang in treating insomnia. In Figure 8. Draw a bar chart of the top 30 key proteins based on their Degree values. In Figure 9. The top 5° are APP, IL-6, TNF, CXCL8, and F2. GO-BP analysis determined that the main biological processes of the target protein include second messenger mediated signaling, regulation of neurotransmitter levels, reactive oxygen species metabolism, and circulatory system in vascular processes. In Figure 10. GO-CC analysis determined that the main cellular components of the target protein include membrane rafts, chloride ion channel complexes, GABA-A receptor complexes, gamma aminobutyric acid receptor complexes, *etc.* In Figure 11. GO-MF analysis determined that the main molecular functions of the target protein include neurotransmitter receptor activity, cytokine receptor binding, neurotransmitter receptor activity involved in regulating postsynaptic membrane potential, neurotransmitter binding, GABA gated ion channel activity, GABA-A receptor activity, GABA receptor activity, benzodiazepine receptor activity, *etc.* In Figure 12. The top 20 signaling pathways closely related to co targets were obtained through KEGG co enrichment analysis, and it was found that the most relevant signaling pathways for insomnia were arranged in order of importance, including the neuroactive ligand receptor interaction signaling pathway, calcium signaling pathway, IL-17 signaling pathway, TNF signaling pathway, Toll like receptor signaling pathway, serotonergic synapse, *etc.* In Figure 13. We focused on the signaling pathway with the highest enrichment of target proteins and the lowest P-value, which is most relevant to the potential mechanism of action of Jiawei Suanzaoren Tang in anti insomnia, namely, the interaction between neuroactive ligands and receptors. In Figure 14.

### 3.7 Changes in body weight of rats

**FIGURE 15:**
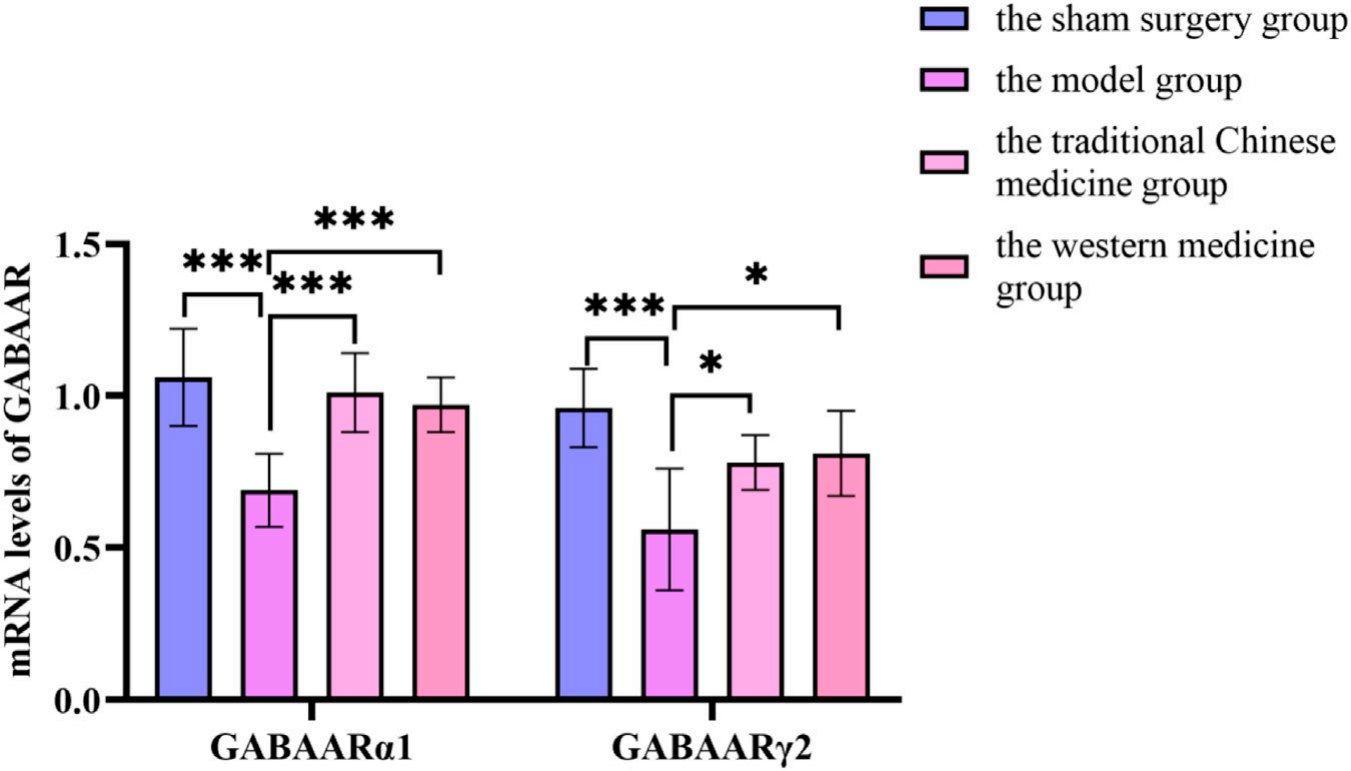
Changes in mRNA levels of GABAA receptor before and after treatment in the sham surgery group, the model group, the traditional Chinese medicine group and the western medicine group.

Compared with before modelling in this group, the weight of rats in the model group, traditional Chinese medicine group and western medicine group decreased significantly (*P* < 0.05). After administration, the weight of rats in the model, traditional Chinese medicine and western medicine groups increased significantly (*P* < 0.05). After administration, the weight of the rats in the Chinese medicine group increased significantly and there was a statistical difference (*P* < 0.05) compared with the rats in the Western medicine group. In Figure 15.

### 3.8 Evaluation of a rat model of perimenopausal insomnia

The SOL of the model group rats was prolonged, the TST was shortened and the sleep rate was reduced, which was significantly different from the sham surgery group (*P* < 0.05). In Table 10. The estrus cycle of rats in the sham surgery group is about 4–5 days, and the vaginal cell smear shows NEC and CEC, showing periodic changes in estrus; the estrus cycle of rats in the model group, traditional Chinese medicine group and western medicine group was disturbed, with prolonged estrus interval and relatively single-cell smear components, with a large amount of white blood cells observed (Supplementary Figure S1). Compared with the sham surgery group, the E_2_ level of the model group rats was significantly decreased, while the FSH and LH levels were significantly increased, with statistical differences (*P* < 0.05). In Supplementary Figure S2.

**TABLE 10:** Sleep status of rats in the sham surgery group and model group.

Group	SOL	TST	Sleep rate (%)
The sham surgery group (n = 10)	7.61 ± 3.83	132.61 ± 20.89	80
The model group (n = 8)	12.61 ± 2.33^*^	102.33 ± 12.76^*^	12.5^*^

The data of SOL and TST were represented by mean ± SD. The sleep rate was expressed as a percentage. *P < 0.05 when compared with the sham surgery group.

### 3.9 Sleep status of rats

The SOL of rats in the Chinese medicine and Western medicine groups was shortened and the TST was prolonged, with significant differences compared with the model group (*P* < 0.05); The SOL of rats in the traditional Chinese medicine group was shortened and the TST was prolonged, with significant differences compared with the Western medicine group (*P* < 0.05). In Supplementary Figure S3.

### 3.10 Changes in E_2_, FSH, LH levels in rat serum

Compared with the model group, the FSH and LH levels in the traditional Chinese medicine group and Western medicine group rats were significantly reduced, while the E_2_ levels were significantly increased, with statistical differences (*P* < 0.05). Compared with the rats in the Western medicine group, the FSH and LH levels in the rats in the traditional Chinese medicine group were significantly reduced, while the E_2_ levels were significantly increased, with statistical differences (*P* < 0.05). In Supplementary Figure S4.

### 3.11 The mRNA levels of 5-HT_1a_ and 5-HT_2a_ receptors in the hypothalamus of rats

Compared with the sham surgery group, the mRNA content of 5-HT_1a_ receptor inthe hypothalamus of rats in the model group was significantly decreased, while the mRNA content of 5-HT_2a_ receptor was significantly increased, with statistical significance (*P* < 0.05). Compared with the model group, the mRNA content of 5-HT_1a_ receptor in the hypothalamus of rats in the traditional Chinese medicine group and the western medicine group was significantly increased, while the mRNA content of 5-HT_2a_ receptor was significantly decreased, with statistical significance (*P* < 0.05). In Supplementary Figure S5.

### 3.12 The mRNA content of GABAAR_α1_ and GABAAR_γ2_ in the hypothalamus of rats

Compared with the sham surgery group, the levels of GABAAR_α1_ and GABAAR_γ2_ in the hypothalamus of the model group rats decreased significantly (*P* < 0.05); compared with the model group, the levels of GABAAR_α1_ and GABAAR_γ2_ in the traditional Chinese medicine group and the western medicine group increased significantly (*P* < 0.05). In Supplementary Figure S6.

## 4 Discussion

This study conducted a 4-week study in patients with perimenopausal insomnia treated with JW-SZRT and lorazepam, comparing the efficacy and safety of JW-SZRT and lorazepam alone in the treatment of perimenopausal insomnia.

Perimenopausal women are very susceptible to sleep and emotional problems. The obvious symptoms of insomnia in perimenopausal women include: sleep disturbances mainly caused by insomnia (Zhao et al., 2022), fever in the hands and feet, sweating during sleep at night (Min et al., 2022), and restlessness in the heart. These symptoms are mostly characterised by yin deficiency and fire excess (Meng et al., 2016), that is, deficiency of yin essence and excess of heart and liver fire. Treatment of perimenopausal insomnia is mainly based on hormone replacement therapy (Dorsey et al., 2004), which may be supplemented by the use of autonomic nervous system modulators such as GABA receptor modulators (Dolev, 2011), low-dose medications suitable for treating depressive symptoms (Soares et al., 2010). Non pharmacological treatment: Pay attention to sleep hygiene, CBT-I treatment, physical therapy, *etc.* Hormone replacement therapy increases the incidence of breast cancer (Katalinic and Rawal, 2008), endometrial cancer and cardiovascular disease in patients, and is associated with significant side effects, so it is not the first choice (Archer, 2001). The sedative-hypnotic drug lorazepam, also known as BZDs, can effectively improve sleep problems in perimenopausal patients and has a good therapeutic effect on conscious symptoms such as anxiety, insufficient sleep time, difficulty falling asleep and easy awakening after sleep.

Traditional Chinese medicine has a good clinical therapeutic effect on perimenopausal insomnia and has fewer side effects and higher safety compared to Western medicine. Research has shown that the total saponins, total brass and alkaloids in Suanzaoren can inhibit the activity of the nervous system to some extent, reduce its activity, have a sedative effect and promote sleep (Stramba-Badiale, 2009); Polysaccharides and flavonoids have a certain degree of alleviating negative emotions such as anxiety and depression, and their effects are closely related to neurotransmitters. Insomnia-related neural receptors have identified the GABAAR as a key target in the treatment of human insomnia. Studies have shown that most of the surface regions of GABAA receptor subunits α1 and γ2 contain binding sites for diazepam and other benzodiazepines (Belelli et al., 2021). The main ingredient of JW-SZRT is Suanzaoren, and a study on the active ingredients of Suanzaoren has shown that it can enhance the expression of GABAAR_α1_ and GABAAR_γ2_, inhibit neuronal excitation, and improve sleep. Its effects are similar to those of the diazepam group (Xiao F et al., 2022). In addition, we are currently in the process of learning *in vivo* electrophysiological techniques with the goal of assessing the EEG power in different sleep stages of insomnia model mice treated with Jiawei Suanzaoren Decoction. To our regret, we are not yet in a position to provide this data immediately. However, this will be a primary focus of our future research efforts, and we plan to dedicate substantial resources to it moving forward.

JW-SZRT can clear the heart and nourish the liver, relieve anxiety and calm the mind. It has a significant therapeutic effect on insomnia of yin deficiency and fire excess type during perimenopause, and can significantly improve the sleep and anxiety status of insomnia patients. Our previous research confirmed the short-term therapeutic effect of JW-SZRT on insomnia patients with anxiety (Gao et al., 2019), and found that after taking JW-SZRT for 4 weeks, sleep quality was significantly improved and anxiety was significantly relieved.

Our research results indicate that JW-SZRT can significantly reduce the PSQI and Kupperman scores of perimenopausal insomnia patients, regulate hormone levels, and improve the conscious symptoms of perimenopausal insomnia patients. Compared with the sedative and hypnotic drug lorazepam, JW-SZRT has a superior therapeutic effect on perimenopausal insomnia patients and is worthy of clinical recommendation. Our research results indicate that JW-SZRT can significantly improve the conscious symptoms of perimenopausal insomnia patients. Compared with lorazepam, JW-SZRThas a superior therapeutic effect on perimenopausal insomnia patients.

There are still many shortcomings and improvements to be made in this clinical study: firstly, the sample size is too small, which may affect the experimental results to some extent; secondly, the research period is not long, especially considering drug tolerance or dependence. Thirdly, the scoring of the scale is very subjective.

Network pharmacology shows that Jiawei Suanzaoren Tang can treat insomnia and achieve neuroprotective effects by regulating the signaling pathway of neuroactive ligand receptor interactions. This theory is also applicable to perimenopausal insomnia. By constructing an animal model of perimenopausal insomnia and monitoring changes in GABAA receptors and 5-HT receptors, the mechanism of Jiawei Suanzaoren Tang in treating perimenopausal insomnia can be revealed at the protein level.

Physical methods for constructing an insomnia model include the enhanced small platform method, exercise method and stimulation; chemical methods such as PCPA, thyroxine, 5-HT7 receptor antagonist, NMDA, *etc.* Before using the enhanced level table sleep deprivation method for modelling, rats must be acclimated to the environment in advance to eliminate the influence of other factors on the experiment. The forced-exercise method can be carried out using appropriate experimental equipment, but the continuous exercise itself may cause the body to enter a state of stress; the stimulation method has a high manpower requirement and cannot be sustained; PCPA modelling is fast and convenient, but the operator must be flexible in controlling the dosage. Sleep experiments can be used to determine whether an insomnia model has been successfully constructed by injecting pentobarbital sodium into the abdominal cavity and comparing sleep parameters such as sleep latency, total sleep time and sleep onset rate in rats after medication.

A normal mature female rat has a sexual cycle of 4–5 days: proestrus, which is the follicular phase, with NEC being the main factor; estrus, which is the ovulatory phase, with CEC being the main factor; in the late estrus, which is the early luteal phase, in addition to the above two types of cells, white blood cells can also be seen; during the estrus phase, which includes the mid to late luteal phase and the early follicular phase, vaginal smears are mainly composed of a large number of white blood cells. Model evaluation criteria: Vaginal smears should be taken from 5 days after surgery. The entire estrous cycle is disrupted and the interval between estrous cycles is significantly prolonged. A large number of white blood cells are seen on the vaginal smear, indicating that the perimenopausal model is well reproduced. It is now common to see a decrease in serum E_2_ levels and an increase in FSH and LH levels (Liu et al., 2015; Randolph et al., 2004).

The hypothalamus is involved in regulating various physiological activities in the body, including sleep, mood, and endocrine status (Li et al., 2004; Luo et al., 2024). The hypothalamus regulates sleep in the ventral lateral preoptic area and after the perimenopause, overall hormone levels are mainly regulated by the hypothalamus-pituitary gland (Arrigoni and Fuller, 2022). In addition, the mRNA of 5-HT_la_R, 5-HT_2a_R, GABAAR_α1_ andGABAAR_γ2_ can be expressed in the hypothalamus (Li et al., 2004), so the hypothalamus was chosen as the research site for this experiment (Grignaschi et al., 1996; Xiao et al., 2022).

The 5-HT receptor is involved in the regulation of sleep and mood, with the highest levels in the brain found in the dorsal raphe nucleus (Monti, 2010). Upstream fibres of the monoamine neurotransmitter 5-HT can be mapped to the hypothalamus via the dorsal raphe nucleus. The expression level of 5-HT receptors is up- or downregulated, closely related to sleep, mainly consisting of 5-HT_la_R and 5-HT_2a_R. Increased expression of the 5-HT_1a_ receptor promotes the generation of action potentials, while increased expression of the 5-HT_2a_ receptor inhibits the generation of action potentials (Lladó-pelford et al., 2012). The regulatory mechanisms of the two on sleep are different. The increased expression level of 5-HT_1a_ receptor can promote the increase of 5-HT content in the brain, making it difficult for the human body to quickly enter the rapid eye movement sleep period, and the overall duration of rapid eye movement sleep is shortened. The 5-HT_2a_ receptor has a stimulating effect on wakefulness, while the expression level of 5-HT_2a_ receptor is reduced, and the duration of slow-wave sleep and non-rapid eye movement sleep is prolonged, thereby improving sleep efficiency and increasing sleep depth.

Women entering the perimenopause experience dysfunction of the HPO axis and reduced oestrogen secretion. There is a large amount of ER_β_ in the central nervous system, it plays an important regulatory role in the synthesis and utilisation of 5-HT in the body. Oestrogen can, to some extent, increase the expression levels of the tryptophan hydroxylase 2 gene and protein, promote the synthesis of the monoamine neurotransmitter 5-HT and increase the body’s need for5-HT transporter (Wang et al., 2022; Charoenphandhu et al., 2011). Oestrogen can also affect the metabolic levels of monoamine oxidase in the body (McLaren et al., 2024), mainly playing a negative regulatory role, causing a relative increase in 5-HT levels, which affects sleep and emotional states. The results of this experiment showed that the level of 5-HT_1a_ receptor mRNA in the hypothalamus of perimenopausal insomnia rats decreased significantly, while the level of 5-HT_2a_ receptor mRNA increased significantly. After treatment with JW-SZRT, the expression level of 5-HT_1a_ receptor mRNA increased, while the expression level of 5-HT_2a_ receptor mRNA decreased. This indicates that JW-SZRT can regulate the expression of 5-HT_1a_ and 5-HT_2a_ receptor subtypes mRNA in the hypothalamus of perimenopausal insomnia rats to achieve the therapeutic effect of insomnia.

The ventrolateral preoptic area of the hypothalamus contains a large number of GABAergic neurons (Saito et al., 2018). GABA and its receptors play important regulatory roles in sleep and emotion. Among them, there are proteins on the cell membrane of the A-type receptors α_1_ and γ_2_ subtypes that promote the transport of CI^−^. After binding to the amino acid neurotransmitter GABA, CI^−^ enters the cell from the outside, causing changes in the potential inside and outside the cell membrane. The cell membrane inhibits the formation of the action potential and promotes the sedative effect of GABA. Elevated GPR30 levels increase the expression levels of amino acid neurotransmitter GABAA receptor genes and proteins, promote the transmission of amino acid neurotransmitter GABA, inhibit excitatory transmission of the nervous system, promote inhibitory transmission of the nervous system, and exert a sedative effect (Liu et al., 2015). In this experiment, the levels of GABAAR_α1_ and GABAAR_γ2_ were significantly decreased in the hypothalamus of perimenopausal insomnia rats, and after treatment with JW-SZRT, the levels of GABAAR_α1_ and GABAAR_γ2_ were significantly increased, indicating to some extent that JW-SZRT can affect the gene expression levels of GABAAR_α1_ and GABAAR_γ2_ in the hypothalamus of perimenopausal insomnia rats, thereby playing a role in regulating the sleep-wake cycle. Due to the diversity and complexity of neurotransmitters and their receptors, the mechanism of action of JW-SZRT on perimenopausal insomnia requires further research.

This animal experiment still has many shortcomings: firstly, short-term sleep deprivation and no long-term treatment and research. Second, there is a lack of behavioural indicators as a reference. Thirdly, due to factors such as funding, only one region of the hypothalamus was selected, with no other regions for comparison and no in-depth research.

## 5 Conclusion

Clinical studies have shown that Jiawei Suanzaoren Decoction can improve the sleep status and quality of life of perimenopausal patients with insomnia caused by yin deficiency and excessive fire, and is worthy of promotion. Network pharmacology has elucidated the relevant targets of Jiawei Suanzaoren Tang in treating insomnia, among which the signaling pathway of neuroactive ligand receptor interaction plays an important role in the treatment of insomnia with Jiawei Suanzaoren Tang. This animal experimental study further indicates that: Firstly, Jiawei Suanzaoren Decoction can prolong the sleep time and sleep duration of perimenopausal insomnia rats and improve the sleep conditions. Secondly, Jiawei Suanzaoren Decoction can cause a decrease in FSH and LH levels and an increase in E2 levels in perimenopausal insomnia rats, and regulate the levels of E2, FSH and LH. Thirdly, JW-SZRT can increase the mRNA gene expression level of 5-HT1a receptor in the hypothalamus of perimenopausal insomnia rats, decrease the mRNA gene expression level of 5-HT2a receptor, and increase the gene expression levels of GABAARα1 and GABAARγ2. This has certain reference significance for further investigation of the mechanism of action of Jiawei Suanzaoren Decoction in the treatment of perimenopausal insomnia. Jiawei Suanzaoren Tang has indeed shown certain therapeutic effects in clinical practice, especially in the treatment of mental and neurological disorders such as insomnia. However, it also has some limitations. Firstly, the causes of insomnia are complex, and everyone’s constitution and condition are different. Therefore, when using Jiawei Suanzaoren Tang, it is necessary to differentiate and treat according to the specific condition of the patient. Secondly, long-term or excessive use of jujube seeds may cause discomfort such as allergies and fatigue. Moreover, when using the modified sour jujube seed soup, attention should also be paid to the interactions between the drugs. If not properly matched, it may lead to adverse reactions. In addition, the modified sour jujube seed soup is limited in clinical application due to its poor taste, limited availability of foreign medicinal materials, difficulty in carrying, and slow onset of action.

## Data Availability

The original contributions presented in the study are included in the article/Supplementary Material, further inquiries can be directed to the corresponding authors.

## References

[B1] Archer D. F. (2001). The effect of the duration of progestin use on the occurrence of endometrial cancer in postmenopausal women. Menopause J. North Am. Menopause Soc. 8, 245–251. 10.1097/00042192-200107000-00005 11449081

[B2] Arrigoni E. Fuller P. M. (2022). The sleep-promoting ventrolateral preoptic nucleus: what have we learned over the past 25 years? Int. J. Mol. Sci. 23, 2905–0067. 10.3390/ijms23062905 35328326 PMC8954377

[B3] Belelli D. Hales T. G. Lambert J. J. Luscher B. Olsen R. Peters J. A. (2021). GABA(A) receptors in GtoPdb v.2021.3. IUPHAR/BPS guide Pharmacol. CITE. 2021, 2633–1020. 10.2218/gtopdb/F72/2021.3 PMC8734037 35005623

[B4] Charoenphandhu J. Teerapornpuntakit J. Nuntapornsak A. Krishnamra N. Charoenphandhu N. (2011). Anxiety-like behaviors and expression of SERT and TPH in the dorsal raphé of estrogen- and fluoxetine-treated ovariectomized rats. Pharmacol. Biochem. Behav. 98, 503–510. 10.1016/j.pbb.2011.02.023 21382399

[B5] Chhibber A. Woody S. K. Karim Rumi M. A. Soares M. J. Zhao L. (2017). Estrogen receptor β deficiency impairs BDNF-5-HT2A signaling in the hippocampus of female brain: a possible mechanism for menopausal depression. Psychoneuroendocrinology 82, 107–116. 10.1016/j.psyneuen.2017.05.016 28544903 PMC5523821

[B6] Dai D. Zheng B. Yu Z. Lin S. Tang Y. Chen M. (2021). Right stellate ganglion block improves learning and memory dysfunction and hippocampal injury in rats with sleep deprivation. BMC Anesthesiol. 21, 272. 10.1186/s12871-021-01486-4 34749669 PMC8574040

[B7] Dellal S. S. Luo R. Otis T. S. (2012). Gabaa receptors increase excitability and conduction velocity of cerebellar parallel fiber axons. J. neurophysiology 107, 2958–2970. 10.1152/jn.01028.2011 22378171 PMC3378368

[B8] Dolev Z. (2011). Case series of perimenopausal women with insomnia treated with mirtazapine followed by prolonged-release melatonin add-on and monotherapy. Archives women's Ment. health 14, 269–273. 10.1007/s00737-011-0205-7 21311927

[B9] Dorsey C. M. Lee K. A. Scharf M. B. (2004). Effect of zolpidem on sleep in women with perimenopausal and postmenopausal insomnia: a 4-week, randomized, multicenter, double-blind, placebo-controlled study. Clin. Ther. 26, 1578–1586. 10.1016/j.clinthera.2004.10.003 15598474

[B10] Gao J. Wang Q. Huang Y. Tang K. Yang X. Cao Z. (2019). *In silico* study of anti-insomnia mechanism for suanzaoren prescription. Front. Pharmacol. 10, 925. 10.3389/fphar.2019.00925 31507421 PMC6713715

[B11] Grignaschi G. Sironi F. Samanin R. (1996). Stimulation of 5-HT2A receptors in the paraventricular hypothalamus attenuates neuropeptide Y-induced hyperphagia through activation of corticotropin releasing factor. Brain Res. 708, 173–176. 10.1016/0006-8993(95)01373-3 8720874

[B12] Hu L. L. Zhang X. Liu W. J. Li M. Zhang Y. H. (2015). Suan zao ren tang in combination with zhi zi chi tang as a treatment protocol for insomniacs with anxiety: a randomized parallel-controlled trial. Evidence-based complementary Altern. Med. eCAM 2015, 913252. 10.1155/2015/913252 PMC4352487 25793006

[B13] Katalinic A. Rawal R. (2008). Decline in breast cancer incidence after decrease in utilisation of hormone replacement therapy. Breast cancer Res. Treat. 107, 427–430. 10.1007/s10549-007-9566-z 17453336

[B14] Li Q. Holmes A. Ma L. Van de Kar L. D. Garcia F. Murphy D. L. (2004). Medial hypothalamic 5-hydroxytryptamine (5-HT)1A receptors regulate neuroendocrine responses to stress and exploratory locomotor activity: application of recombinant adenovirus containing 5-HT1A sequences. J. Neurosci. official J. Soc. Neurosci. 24, 10868–10877. 10.1523/JNEUROSCI.3223-04.2004 PMC6730203 15574737

[B15] Li Y. Xu L. Qin Z. (2014). Effects of electroacupuncture stimulation of “sanyinjiao” (sp 6) on hypothalamus'-pituitary-ovary axis in perimenopausal rats. Acupunct. Res. 39, 198–201. 25069195

[B16] Liao J. F. Jan Y. M. Huang S. Y. Wang H. H. Yu L. L. Chen C. F. (1995). Evaluation with receptor binding assay on the water extracts of ten CNS-active Chinese herbal drugs. Proc. Natl. Sci. Counc. Repub. China Part B Life Sci. 3, 151–158. 7480361

[B17] Liu S. B. Tian Z. Guo Y. Y. Zhang N. Feng B. Zhao M. G. (2015). Activation of GPR30 attenuates chronic pain-related anxiety in ovariectomized mice. Psychoneuroendocrinology 53, 94–107. 10.1016/j.psyneuen.2014.12.021 25614360

[B18] Liu W. Wang L. Y. Xing X. X. Fan G. W. (2015). Conditions and possible mechanisms of VCD-induced ovarian failure. Altern. laboratory animals ATLA 43, 385–392. 10.1177/026119291504300606 26753941

[B19] Lladó-Pelfort L. Santana N. Ghisi V. Artigas F. Celada P. (2012). 5-HT1A receptor agonists enhance pyramidal cell firing in prefrontal cortex through a preferential action on GABA interneurons. Cereb. cortex 22, 1487–1497. 10.1093/cercor/bhr220 21893679

[B20] Luo Y. Yu L. Zhang P. Lin W. Xu H. Dou Z. (2024). Larger hypothalamic subfield volumes in patients with chronic insomnia disorder and relationships to levels of corticotropin-releasing hormone. J. Affect. Disord. 351, 870–877. 10.1016/j.jad.2024.02.023 38341156

[B21] McLaren S. Seidler K. Neil J. (2024). Investigating the role of 17β-estradiol on the serotonergic system, targeting soy isoflavones as a strategy to reduce menopausal depression: a mechanistic review. J. Am. Nutr. Assoc. 43, 221–235. 10.1080/27697061.2023.2255237 37695875

[B22] Meng F. Duan P. Hu Q. Wang Y. Wang Q. Zhang M. (2016). Scrapping therapy combined with Qingxin Zishen Decoction for perimenopausal syndrome with pattern of fire excess from yin deficiency. Zhongguo Zhen Jiu 36, 821–826. 10.13703/j.0255-2930.2016.08.012 29231567

[B23] Min S. H. Yang Q. Docherty S. L. Im E. O. Hu X. (2022). Symptom clusters and key symptoms among midlife perimenopausal and postmenopausal women with and without metabolic syndrome. Nurs. Res. 71, E28–E38. 10.1097/NNR.0000000000000591 35759720 PMC9237449

[B24] Monti J. M. (2010). The role of dorsal raphe nucleus serotonergic and non-serotonergic neurons, and of their receptors, in regulating waking and rapid eye movement (REM) sleep. Sleep. Med. Rev. 14, 319–327. 10.1016/j.smrv.2009.10.003 20153670

[B25] Monti J. M. Jantos H. (2003). Differential effects of the 5-HT1A receptor agonist flesinoxan given locally or systemically on rem sleep in the rat. Eur. J. Pharmacol. 478, 121–130. 10.1016/j.ejphar.2003.08.039 14575796

[B26] Pei-Lu Y. Chon-Haw T. Ya-Chu C. (2007). Gamma-aminobutyric acid (GABA) receptor mediates suanzaorentang, a traditional Chinese herb remedy, -induced sleep alteration. J. Biomed. Sci. 2, 285–297. 10.1007/s11373-006-9137-z 17151826

[B27] Randolph J. F. Jr Sowers M. Bondarenko I. V. Harlow S. D. Luborsky J. L. Little R. J. (2004). Change in estradiol and follicle-stimulating hormone across the early menopausal transition: effects of ethnicity and age. J. Clin. Endocrinol. metabolism 89, 1555–1561. 10.1210/jc.2003-031183 15070912

[B28] Sá S. I. Fonseca B. M. Teixeira N. Madeira M. D. (2015). Estrogen receptors α and β have different roles in the induction and trafficking of progesterone receptors in hypothalamic ventromedial neurons. FEBS J. 282, 1126–1136. 10.1111/febs.13207 25612677

[B29] Saito Y. C. Maejima T. Nishitani M. Hasegawa E. Yanagawa Y. Mieda M. (2018). Monoamines inhibit GABAergic neurons in ventrolateral preoptic area that make direct synaptic connections to hypothalamic arousal neurons. J. Neurosci. official J. Soc. Neurosci. 38, 6366–6378. 10.1523/JNEUROSCI.2835-17.2018 PMC6596100 29915137

[B30] Sakurai T. (2012). Hypothalamic neuropeptides implicated in the regulation of sleep/wakefulness states. Brain Nerve 64, 629–637. 22647470

[B31] Shi X. L. Zhao C. Yang S. Hu X. Y. Liu S. M. (2015). Moxibustion reduces ovarian granulosa cell apoptosis associated with perimenopause in a natural aging rat model. Evidence-based complementary Altern. Med. eCAM. 2015, 742914. 10.1155/2015/742914 PMC4621346 26550020

[B32] Soares C. N. Frey B. N. Haber E. Steiner M. (2010). A pilot, 8-week, placebo lead-in trial of quetiapine extended release for depression in midlife women: impact on mood and menopause-related symptoms. J. Clin. Psychopharmacol. 30, 612–615. 10.1097/JCP.0b013e3181f1d0f2 20814317

[B33] Stramba-Badiale M. (2009). Postmenopausal hormone therapy and the risk of cardiovascular disease. J. Cardiovasc. Med. 10, 303–309. 10.2459/JCM.0b013e328324991c 19430340

[B34] Tian Z. Wang Y. Zhang N. Guo Y. Y. Feng B. Liu S. B. (2013). Estrogen receptor gpr30 exerts anxiolytic effects by maintaining the balance between gabaergic and glutamatergic transmission in the basolateral amygdala of ovariectomized mice afterstress. Psychoneuroendocrinology 38, 2218–2233. 10.1016/j.psyneuen.2013.04.011 23669322

[B35] Wang B. Shi H. Ren L. Miao Z. Wan B. Yang H. (2022). Ahi1 regulates serotonin production by the GR/ERβ/TPH2 pathway involving sexual differences in depressive behaviors. Cell. Commun. Signal. CCS 20, 74. 10.1186/s12964-022-00894-4 35643536 PMC9148486

[B36] Xiao F. Shao S. Zhang H. Li G. Piao S. Zhao D. (2022). Neuroprotective effect of Ziziphi Spinosae Semen on rats with p-chlorophenylalanine-induced insomnia via activation of GABA(A) receptor. Front. Pharmacol. 13, 965308. 10.3389/fphar.2022.965308 36483742 PMC9722729

[B37] Xiao F. Shao S. Zhang H. Li G. Piao S. Zhao D. (2022). Neuroprotective effect of Ziziphi Spinosae Semen on rats with p-chlorophenylalanine-induced insomnia via activation of GABA(A) receptor. Front. Pharmacol. 13, 965308. 10.3389/fphar.2022.965308 36483742 PMC9722729

[B38] Zhang Q. Zhang M. Liu Y. Wang Y. Lv F. Wang Y. (2023). Exploring the therapeutic mechanism of Liuwei Suanzao decoction for perimenopausal insomnia based on network pharmacology and animal experiments. Nan Fang. Yi Ke Da Xue Xue Bao 43, 1536–1547. 10.12122/j.issn.1673-4254.2023.09.11 37814868 PMC10563099

[B39] Zhao Y. Niu H. Liu S. (2022). Effects of aerobics training on anxiety, depression and sleep quality in perimenopausal women. Front. psychiatry 13, 1025682. 10.3389/fpsyt.2022.1025682 36506429 PMC9730414

