# Supplementary material for: Jiawei Suanzaoren decoction for the treatment of perimenopausal insomnia: clinical observation and experimental study
Source: Front Pharmacol. 2025 Jan 29;15:1495957. doi: 10.3389/fphar.2024.1495957 (PMC11813739; doi:10.3389/fphar.2024.1495957)
Supplement: Supplementary file 1 [file DataSheet1.zip › Suppl. Mat. Fig.4.pdf]

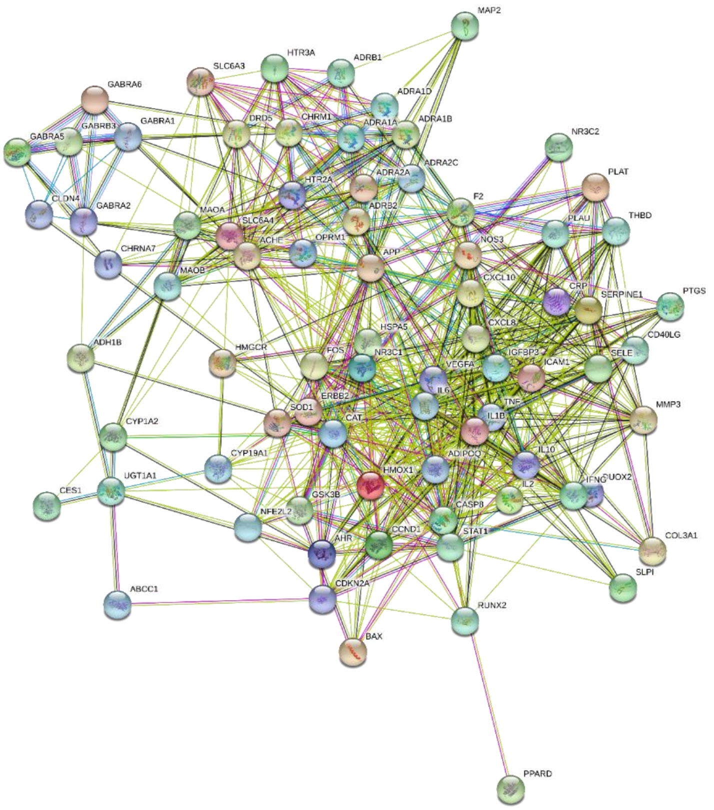

Suppl. Mat. Fig.4. PPI network diagram of the interaction between the active ingredients and targets of Jiawei Suanzaoren Decoction in treating insomnia
